# Essential Role for Endogenous siRNAs during Meiosis in Mouse Oocytes

**DOI:** 10.1371/journal.pgen.1005013

**Published:** 2015-02-19

**Authors:** Paula Stein, Nikolay V. Rozhkov, Fan Li, Fabián L. Cárdenas, Olga Davydenk, Lee E. Vandivier, Brian D. Gregory, Gregory J. Hannon, Richard M. Schultz

**Affiliations:** 1 Department of Biology, University of Pennsylvania, Philadelphia, Pennsylvania, United States of America; 2 Cold Spring Harbor Laboratory, Watson School of Biological Sciences and Howard Hughes Medical Institute, Cold Spring Harbor, New York, United States of America; 3 CRUK Cambridge Institute, Li Ka Shing Centre, University of Cambridge, Cambridge, United Kingdom; Cornell University, UNITED STATES

## Abstract

The RNase III enzyme DICER generates both microRNAs (miRNAs) and endogenous short interfering RNAs (endo-siRNAs). Both small RNA species silence gene expression post-transcriptionally in association with the ARGONAUTE (AGO) family of proteins. In mammals, there are four AGO proteins (AGO1-4), of which only AGO2 possesses endonucleolytic activity. siRNAs trigger endonucleolytic cleavage of target mRNAs, mediated by AGO2, whereas miRNAs cause translational repression and mRNA decay through association with any of the four AGO proteins. *Dicer* deletion in mouse oocytes leads to female infertility due to defects during meiosis I. Because mouse oocytes express both miRNAs and endo-siRNAs, this phenotype could be due to the absence of either class of small RNA, or both. However, we and others demonstrated that miRNA function is suppressed in mouse oocytes, which suggested that endo-siRNAs, not miRNAs, are essential for female meiosis. To determine if this was the case we generated mice that express a catalytically inactive knock-in allele of *Ago2 (Ago2ADH)* exclusively in oocytes and thereby disrupted the function of siRNAs. Oogenesis and hormonal response are normal in *Ago2ADH* oocytes, but meiotic maturation is impaired, with severe defects in spindle formation and chromosome alignment that lead to meiotic catastrophe. The transcriptome of these oocytes is widely perturbed and shows a highly significant correlation with the transcriptome of *Dicer* null and *Ago2* null oocytes. Expression of the mouse transcript (MT), the most abundant transposable element in mouse oocytes, is increased. This study reveals that endo-siRNAs are essential during meiosis I in mouse females, demonstrating a role for endo-siRNAs in mammals.

## Author Summary

In animals, the three main classes of small RNAs are microRNAs, short interfering RNAs, and PIWI-interacting RNAs. All three RNA species silence gene expression post-transcriptionally through interaction with the ARGONAUTE family of proteins. In mammals in particular, microRNAs are ubiquitously expressed, are essential for development, and perform numerous functions in a variety of cells and tissues. piRNAs are expressed almost exclusively in the germline, and are essential for male fertility and defense against transposons. Endogenous siRNAs are only expressed in germ cells and embryonic stem cells and have not been ascribed a functional role. By engineering a mouse that expresses a modified ARGONAUTE protein, we disrupt the function of endo-siRNAs exclusively in oocytes and find that females are infertile. Oocytes with an impaired siRNA pathway fail to complete meiosis I, and display severe spindle formation and chromosome alignment defects. Their transcriptome is widely perturbed and expression of the most abundant transposon is increased. These findings indicate that endo-siRNAs are essential for female fertility in mouse, are required for spindle formation, chromosome congression, and defense against transposons. This study unequivocally demonstrates an essential function for siRNAs in mammals, mediated through endonucleolytic cleavage of targets, and provides an explanation for the selective pressure that one AGO protein retains catalytic activity.

## Introduction

The RNase III enzyme DICER is responsible for biosynthesis of short-interfering RNAs (siRNAs) and microRNAs (miRNAs). DICER processes long double-stranded RNA (dsRNA) precursors into 21–23 bp-long duplexes known as siRNAs [1]. miRNAs are encoded by specific genomic loci and are processed from endogenous hairpin-shaped transcripts that are initially cleaved in the nucleus to a 70-bp miRNA precursor (pre-miRNA) by the Microprocessor complex, which is composed of the RNase III enzyme DROSHA and its partner, DiGeorge syndrome critical region 8 (DGCR8). The pre-miRNA is exported to the cytoplasm, where DICER cleaves the loop region of the molecule to generate the mature miRNA duplex [2].

Although both siRNAs and miRNAs are synthesized as duplexes, only one of the two strands, the ‘guide’ strand, is incorporated into the multi-protein complex RNA-induced silencing complex (RISC); the other strand (‘passenger’ strand) is discarded [3]. The guide strand recognizes a target mRNA by Watson-Crick base pairing and, based on the degree of sequence complementarity between the small RNA and target mRNA, either endonucleolytic cleavage or translational repression of the target mRNA follows [4]. In animals, siRNAs are perfectly complementary to their targets, and hence trigger mRNA cleavage, whereas miRNAs are usually only partially complementary and silence gene expression by translational repression and mRNA decay. Although it was initially postulated that mRNA levels did not change substantially in response to animal miRNAs, it was later shown that mRNA destabilization, prompted by deadenylation and decapping by the mRNA degradation machinery, is the main mode of regulation by mammalian miRNAs [5]. ARGONAUTE (AGO) proteins are at the core of RISC. In mammals, there are four AGO proteins (AGO1–4). All four can bind small RNAs and trigger translational repression, but only AGO2 possesses endonucleolytic activity and is the catalytic component of RISC [6].

We previously demonstrated a role for small RNAs during meiosis in mouse oocytes. Mice with an oocyte-specific deletion of *Dicer* are infertile due to defects during meiosis I [7,8]. *Dicer*-deficient females have morphologically normal ovaries and oocytes, produce normal numbers of oocytes, and ovulate similar numbers of eggs. However, *Dicer* null oocytes display meiotic catastrophe, with multiple disorganized meiotic spindles and severe chromosome congression defects. Expression of a subset of transposable elements is increased and the transcriptome is widely perturbed in *Dicer* null oocytes, with 18.4% of transcripts mis-regulated [7].

Deep sequencing of small RNAs demonstrated the presence of DICER-dependent miRNAs and endogenous siRNAs (endo-siRNAs), as well as DICER-independent PIWI interacting RNAs (piRNAs) in mouse oocytes [9,10]. Two populations of endo-siRNAs were found, one that corresponds to transposon-rich loci and another that maps to protein-coding genes. Interestingly, we found that some siRNAs are processed from dsRNAs formed by hybridization of transcripts from protein-coding genes to antisense transcripts from homologous pseudogenes and that these endo-siRNAs regulate the expression of endogenous genes. Therefore, the phenotype of *Dicer* null oocytes could be due to the absence of miRNAs or endo-siRNAs, or both. Using mRNA reporters, we assayed the ability of miRNAs to silence gene expression, looking at both translational repression and transcript levels. We found that miRNA activity decreases during oocyte growth and is suppressed in the full-grown oocyte. Furthermore, the very modest translational repression observed is not accompanied by message degradation [11]. Similarly, Suh et al. generated an oocyte-specific deletion of *Dgcr8* and found that *Dgcr8* null oocytes, which lack mature miRNAs, have a normal transcriptome and undergo normal meiotic maturation, fertilization, and embryonic development; consistent with these findings, *Dgcr8* null mice have no discernable phenotype [12]. These two studies suggest that most likely endo-siRNAs, and not miRNAs, have an essential role during female meiosis.

It has recently been reported that mouse oocytes express a truncated DICER isoform, DICER^O^, which lacks the N-terminal DExD helicase domain, and which processes long dsRNAs much more efficiently than the somatic DICER isoform (DICER^S^), which is also expressed, albeit at lower levels [13]. The phenotype of *Dicer* ^*O*^ null mice is virtually identical to the phenotype of mice with an oocyte-specific deletion of *Dicer* (which lack both DICER^S^ and DICER^O^). Although DICER^O^ can produce both miRNAs and endo-siRNAs when ectopically expressed in embryonic stem (ES) cells, miRNA levels appear slightly increased in *Dicer* ^*O*^ null oocytes, suggesting that likely siRNAs are responsible for the observed phenotype. Whether this role of endo-siRNAs is mediated by endonucleolytic cleavage of mRNA targets remains unknown.

To test directly the role of endo-siRNAs through endonucleolytic cleavage in mouse oocytes, we expressed a catalytically inactive knock-in allele of *Ago2* specifically in oocytes to disrupt the function of endo-siRNAs. We find that female mice expressing a catalytically inactive AGO2 (but not active AGO2) in their oocytes are infertile due to meiosis I defects. The phenotype is virtually identical to that in *Dicer* null females—female sterility, defects in spindle formation and chromosome congression, increase in abundance of transposable elements, and widespread changes in the transcriptome—and using live cell imaging, we characterize in more detail the meiotic defects. This study demonstrates a functional role for endogenous siRNAs through endonucleolytic cleavage in mammals and adds support to the evolutionary pressure to conserve ARGONAUTE endonucleolytic activity in animals.

## Results and Discussion

### Generation and characterization of an oocyte-specific catalytically inactive *Ago2* allele

To eliminate the function of siRNAs we generated mice carrying a catalytically inactive form of AGO2 in their oocytes using a knock-in allele of *Ago2* in which the catalytic DDH motif was mutated to ADH (*Ago2* ^ADH^) [14]. This mutation inhibits endonucleolytic cleavage without affecting small RNA binding [6]. However, because mice carrying this allele die shortly after birth, we utilized a breeding scheme using *Ago2* ^ADH^ mice, *Ago2* ^fl/fl^ mice, and mice expressing Cre recombinase driven by the oocyte-specific *Zp3* promoter to produce *Ago2* ^fl/ADH^; Cre/+ females, referred to as *Ago2* ^ADH^ (S1 Fig.). These crosses also generated *Ago2* null mice.

**Fig 1:**
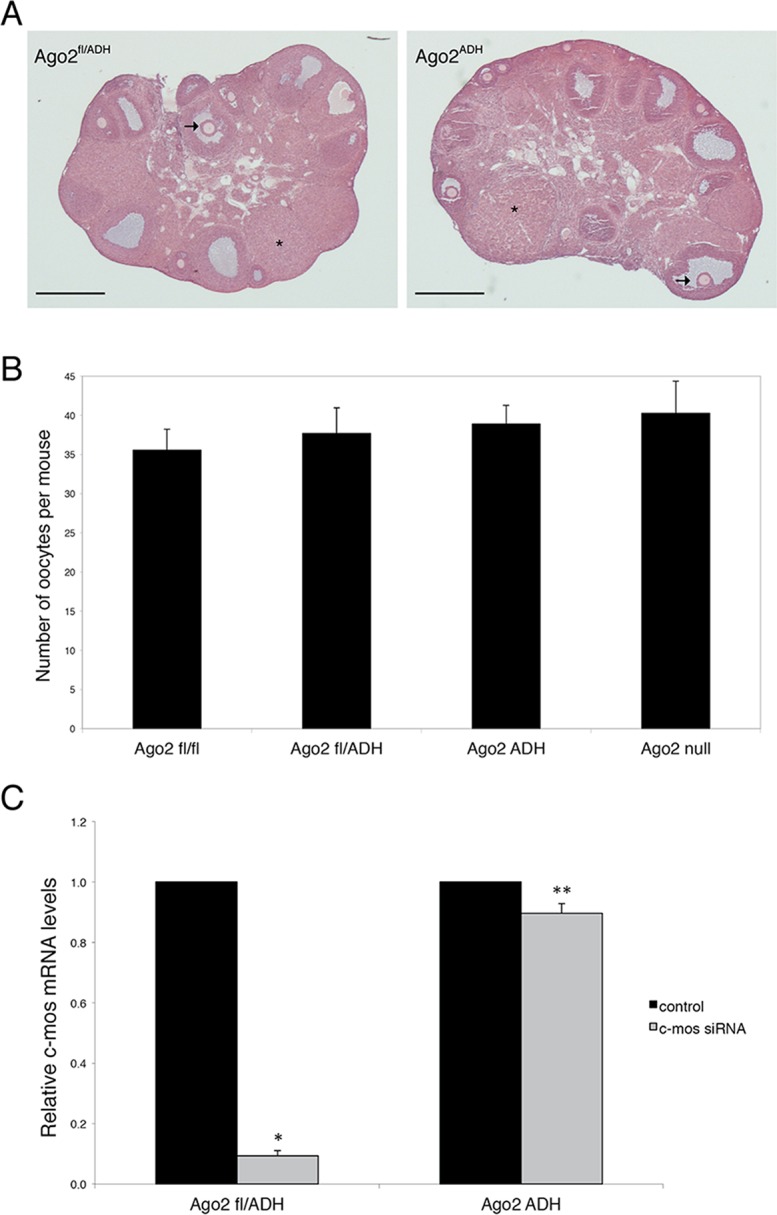
AGO2 catalytic activity is not required for oocyte growth and hormonal response. A) Histological sections of ovaries derived from *Ago2* ^fl/ADH^ (left panel) and *Ago2* ^ADH^ (right panel) females. Hematoxylin and eosin staining was performed as described in Materials and Methods. There were no obvious differences in ovary size, number of follicles, or follicular stages present between the two groups. The arrows indicate antral follicles, whereas the asterisks denote corpora lutea. Scale bar: 500 μm. B) Number of full-grown oocytes recovered from *Ago2* ^fl/fl^, *Ago2* ^fl/ADH^, *Ago2* ^ADH^, and *Ago2* null females. Oocyte collection after equine chorionic gonadotropin (eCG) priming was performed as described in Materials and Methods. The data are presented as the mean ± SEM; 29 *Ago2* ^fl/fl^, 26 *Ago2* ^fl/ADH^, 54 *Ago2* ^ADH^, and 19 *Ago2* null females were utilized. One-way ANOVA was used to compare the different groups and no statistical differences were found. C) Major reduction in AGO2 catalytic activity in oocytes from *Ago2* ^ADH^ mice. Full-grown oocytes were microinjected with *c-Mos* siRNA and *c-Mos* transcript levels were assayed by qRT-PCR 40 h later. The experiment was performed 3 times and statistical analysis was done using one-way ANOVA, followed by Bonferroni post-test. *p<0.001; **p< 0.05.

Ovarian morphology in *Ago2* ^ADH^ females was normal, with follicles at different stages of development, as well as corpora lutea, indicating that ovulation had occurred (Fig. 1A). After hormone stimulation, *Ago2* ^ADH^ females yielded similar numbers of full-grown oocytes as their wild-type (*Ago2* ^fl/fl^) or heterozygous (*Ago2* ^fl/ADH^) counterparts; similar numbers were also present in *Ago2* null females (Fig. 1B). This result indicated that siRNA function is not required for oocyte development or response to hormones. However, *Ago2* ^ADH^ females were unable to produce any offspring during a 6-month mating trial with several wild-type males, indicating female sterility.

To ascertain if oocytes carrying an *Ago2* ^ADH^ allele are incapable of endonucleolytic cleavage of small RNA targets, an RNAi assay was performed with *Ago2* ^ADH^ females. Full-grown oocytes were microinjected with *c-Mos* siRNA and 40 h later *c-Mos* mRNA levels were quantified by qRT-PCR. Whereas oocytes derived from *Ago2* ^fl/fl^ or *Ago2* ^fl/ADH^ females exhibited ~90% decrease in *c-Mos* transcript levels in *c-Mos* siRNA-treated oocytes compared to control oocytes, oocytes obtained from *Ago2* ^ADH^ females only showed a mild reduction (~10%) in *c-Mos* levels (Fig. 1C). These results demonstrated that oocytes from *Ago2* ^ADH^ females had extremely reduced AGO2 catalytic activity. This residual endonucleolytic activity may be due to persistent wild-type AGO2 levels that were present prior to Cre excision, because both mRNAs and proteins are often stable in oocytes.

### AGO2 catalytic activity is required for completion of meiosis in mouse oocytes

**Fig 2:**
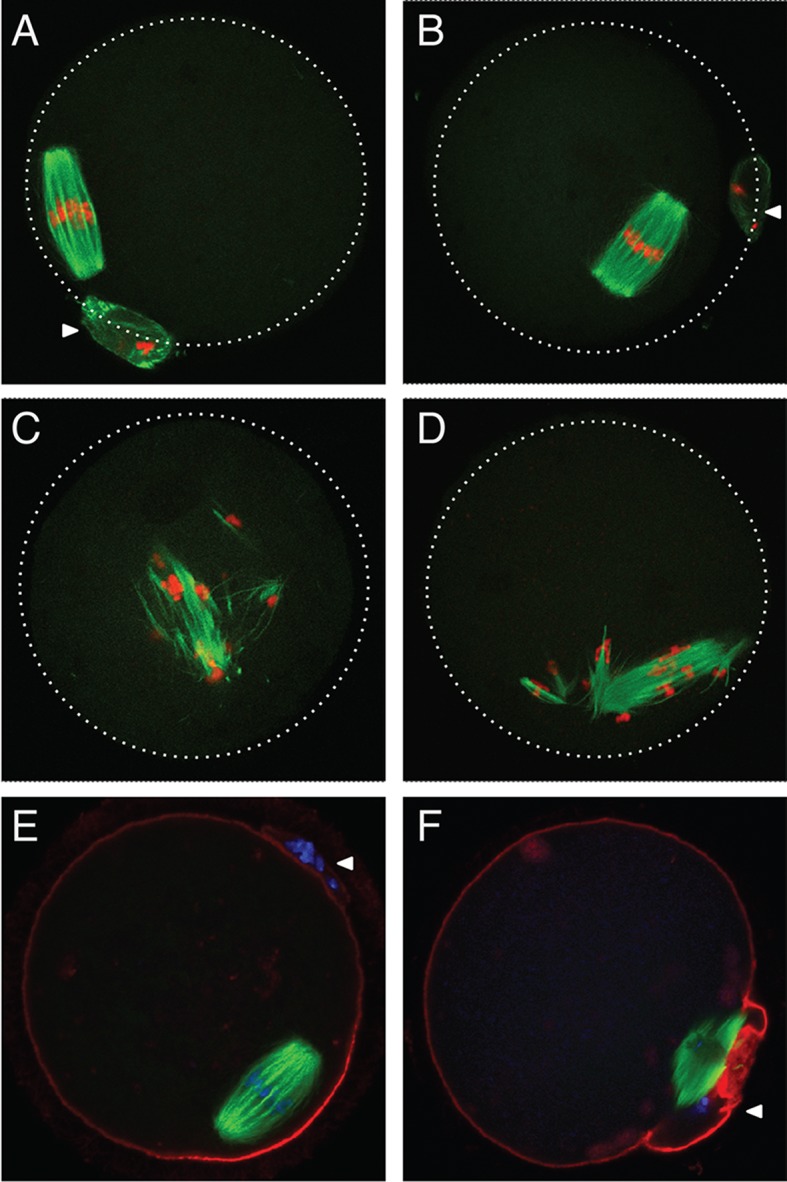
Abnormal meiotic spindles in *Ago2* ^ADH^ oocytes. Oocytes from *Ago2* ^fl/fl^, *Ago2* ^fl/ADH^, *Ago2* ^ADH^, and *Ago2* null females were *in vitro* matured for 16 h. Immunofluorescence was performed as described in Materials and Methods. Arrowheads indicate the first polar body. A-D) Microtubules were stained with an antibody against (-tubulin (green) and DNA was counterstained with TO-PRO3 (red). A) *Ago2* ^fl/fl^ oocyte, B) *Ago2* ^fl/ADH^ oocyte, C) *Ago2* ^ADH^ oocyte, D) *Ago2* null oocyte. E, F) Oocytes from *Ago2* ^fl/ADH^ (E) and *Ago2* ^ADH^ (F) females were stained with an antibody against (-tubulin (green), F-actin was labeled with Alexa 633-conjugated phaloidin (red), and DNA was counterstained with DAPI (blue).

To assess if AGO2 catalytic activity was required for meiotic maturation, full-grown oocytes were *in vitro* matured and spindle morphology was determined by immunofluorescence. Oocytes derived from *Ago2* ^fl/fl^ (Fig. 2A) or *Ago2* ^fl/ADH^ (Fig. 2B) females matured normally to metaphase II, as evidenced by the barrel-shaped meiotic spindle and extrusion of the first polar body. However, oocytes collected from *Ago2* ^ADH^ (Fig. 2C) or *Ago2* null (Fig. 2D) females exhibited abnormal, disorganized spindles, with unaligned chromosomes. Some oocytes derived from *Ago2* ^ADH^ females extruded a polar body; nevertheless, upon closer examination it became clear that meiotic maturation was also abnormal in these oocytes, because partitioning of chromosomes between egg and polar body had not faithfully occurred (Fig. 2E, F).

**Fig 3:**
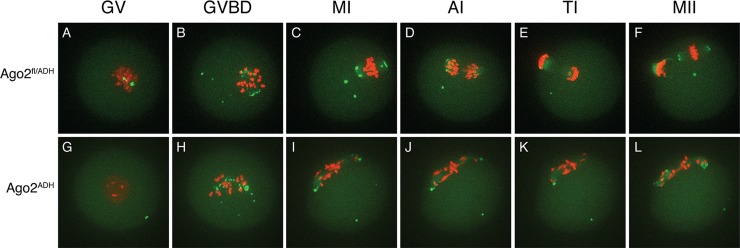
Abnormal chromosome segregation and spindle assembly in *Ago2* ^ADH^ oocytes. Chromosome and spindle dynamics in oocytes expressing AURKA-EGFP (green) and H2B-mCherry (red) were observed by time-lapse live confocal microscopy. Frames at the indicated stages of meiotic maturation were selected from the original time series (S1–S2 Movies), in which images were acquired every 18 min for 16 h. All images are maximal intensity projections of a confocal z series. A-F: *Ago2* ^fl/ADH^ oocytes; G-L: *Ago2* ^ADH^ oocytes. GV: germinal vesicle intact (A, G), GVBD: germinal vesicle breakdown (B, H), MI: metaphase I (C, I), AI: anaphase I (D, J), TI: telophase I (E, K), MII: metaphase II (F, L). The experiment was performed 3 times using at least 10 oocytes per group. Representative images are shown.

To characterize better the meiotic defects, oocytes were microinjected with cRNAs encoding Aurora kinase A (AURKA) fused to EGFP (to label spindle poles) and histone H2B fused to mCherry (to label chromosomes) and live imaging was performed during meiotic maturation (S1–S3 Movies). In *Ago2* ^fl/fl^ or *Ago2* ^fl/ADH^ oocytes (S1 Movie, Fig. 3A-B), the chromosomes remained centrally located and formed a sphere right after germinal vesicle breakdown (GVBD). In contrast, in *Ago2* ^ADH^ oocytes (S2 Movie, Fig. 3G-H), the chromosomes did not congress and instead scattered, covering a large area of the oocyte. *Ago2* ^fl/ADH^ oocytes proceeded to form a barrel-shaped metaphase I spindle, with chromosomes tightly aligned at the metaphase plate (Fig. 3C). Homologous chromosomes then separated at anaphase I (Fig. 3D), and migrated to opposite poles at telophase I (Fig. 3E), followed by cytokinesis, resulting in extrusion of the first polar body, completion of meiosis I and arrest at the metaphase stage of meiosis II (Fig. 3F). In contrast, in most *Ago2* ^ADH^ oocytes, the chromosomes remained dispersed and never aligned, and oocytes failed to enter anaphase I (Fig. 3G-L, S2 Movie). In a few *Ago2* ^ADH^ oocytes, after an initial dispersion of the chromosomes at GVBD, most chromosomes managed to align and form a metaphase I spindle, but there were always a few misaligned chromosomes, which resulted in a failure to enter anaphase and dispersion of chromosomes (S3 Movie).

The severe spindle defects observed in *Dicer* null oocytes have also been described in *Ago2* null oocytes [15]. Although in the latter study the defect was attributed to reduced levels of miRNAs, it was later demonstrated that oocytes devoid of miRNAs have normal meiotic spindles [12]. By utilizing an allele of *Ago2* that can bind small RNAs, but is catalytically inactive, we show that spindle formation and chromosome congression depend on the action of endo-siRNAs. Live imaging technology revealed that the defects start during GVBD, when chromosomes and microtubule organizing centers (MTOCs) scatter instead of forming a sphere [16], resulting in a long, abnormal spindle with unaligned chromosomes that fail to progress to anaphase I. The mechanism that links siRNAs with the spindle defects remains unknown. Given that the transcriptome of *Ago2* ^ADH^ oocytes is widely perturbed (see below), it is unlikely that a single protein is responsible for this phenotype.

### Increase in MT retrotransposon levels in Ago2^ADH^ oocytes

**Fig 4:**
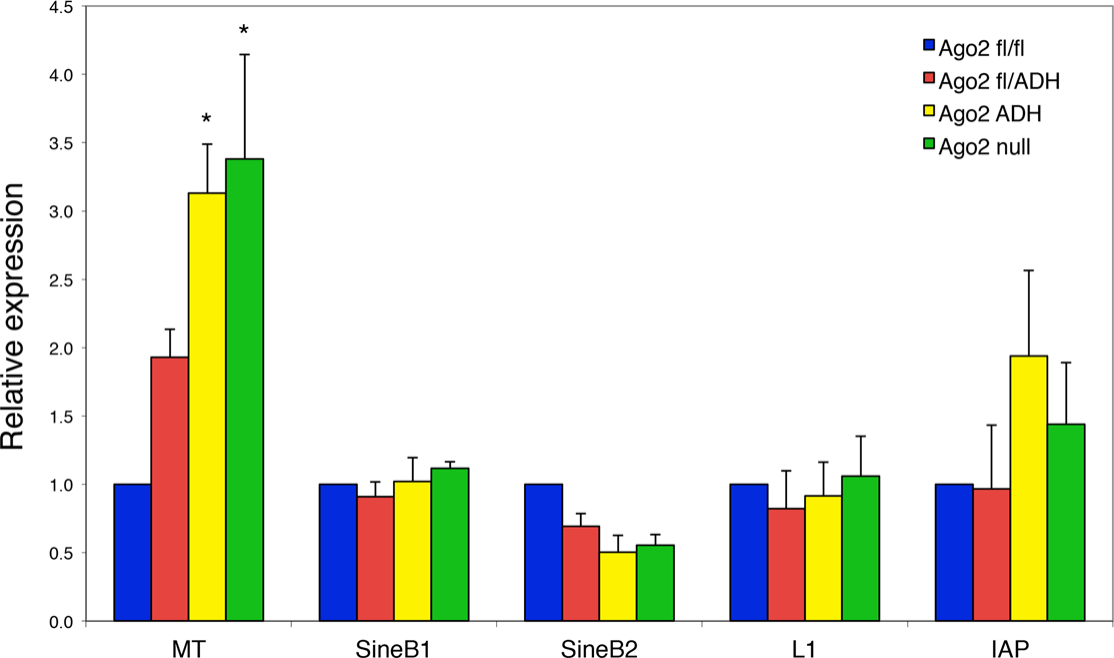
Increased abundance of mouse transcript (MT) retrotransposon in *Ago2* ^ADH^ oocytes. The levels of various transposons were determined by qRT-PCR in oocytes from different *Ago2* genotypes, as described in Materials and Methods. Transposon levels in *Ago2* ^fl/fl^ oocytes were set as 1. Data are expressed as the mean ± SEM of four experiments. *p< 0.05 vs. *Ago2* ^fl/fl^; two-way ANOVA, followed by Bonferroni post-test.

Because the levels of a subset of transposons are increased in *Dicer*-deficient oocytes [7,12], we investigated if this was also the case in the absence of AGO2 catalytic activity. Quantitative RT-PCR of the most abundant transposons in mouse oocytes revealed a significant increase in the levels of mouse transcript (MT), a member of the MaLR family of non-autonomous retrotransposons, in *Ago2* ^ADH^ and *Ago2* null oocytes (Fig. 4). No significant differences were observed for the short interspersed repetitive elements (SINEs), long interspersed repetitive element 1 (LINE1 or L1), or intracisternal A-particle (IAP). This result differs somewhat from what we had previously described in *Dicer* null oocytes, where not only MT, but also Sine B1 and B2 elements were increased. This difference is likely due to differences in genetic background. We found that after re-deriving the *Dicer* null line, only MT levels were increased in oocytes (S2 Fig.), in agreement with a previous study [12], with *Dicer* ^*O*^ null mice [13], and with *Ago2* ^ADH^ oocytes.

PIWI family mutants are male sterile, but female fertile in mouse, indicating that the piRNA system is not essential during oogenesis [17]. This female fertility is not the case in flies and fish, where mutants that disrupt the piRNA system are female sterile [17]. The presence of endo-siRNAs that map to transposons in mouse oocytes likely explains why piRNAs are not essential in females, because both piRNAs and endo-siRNAs repress transposable elements in mouse oocytes. Because MT transcripts account for ~13% of all transcripts in the oocyte [18], a 3-fold increase in abundance is substantial and emphasizes the importance of siRNA action through endonucleolytic cleavage in transposon control.

### Widespread changes in the oocyte transcriptome in the absence of AGO2 catalytic activity

*Dicer*-deficient oocytes exhibit dramatic changes in their transcriptome, as assayed by microarray analysis, with thousands of transcripts up- and down-regulated compared to wild-type oocytes [7,12]. To determine if the same molecular phenotype exists in the absence of AGO2 catalytic activity, we performed high-throughout RNA sequencing (RNA-seq) in full-grown *Ago2* ^fl/fl^, *Ago2* ^ADH^, and *Ago2* null oocytes, as well as *Dicer* wild-type (WT) and knockout (KO) oocytes. We found extensive changes in transcript levels in *Ago2* ^ADH^ and *Ago2* null oocytes. Using a false discovery rate (FDR) of 1%, 6441 transcripts were mis-regulated in *Ago2* ^ADH^ vs. *Ago2* ^fl/fl^ oocytes (3199 up-regulated and 3242 down-regulated) and 6142 transcripts were mis-regulated in *Ago2* null vs. *Ago2* ^fl/fl^ oocytes (3050 up-regulated, 3092 down-regulated). Similarly, 6767 transcripts were mis-regulated in *Dicer* KO vs. WT oocytes (3195 up-regulated, 3572 down-regulated). Interestingly, although similar numbers of transcripts were down-regulated and up-regulated, as we had described for *Dicer* null oocytes, when the dataset was filtered by fold-change, a different picture surfaced. Of those transcripts whose abundance changed at least two-fold, the percentages that were up-regulated vs. down-regulated were 69%/31% in *Ago2* ^ADH^ vs. *Ago2* ^fl/fl^ oocytes, 68%/32% in *Ago2* null vs. *Ago2* ^fl/fl^, and 62%/38% in *Dicer* KO vs. WT oocytes. This finding indicates that the magnitude of change is greater in those transcripts that are up-regulated. This is indeed the case, as shown in S3 Fig., where the absolute values of fold-changes for the different comparisons were plotted for up-regulated and down-regulated transcripts. Because *Cre*-mediated recombination to excise the floxed allele of *Ago2* and impair endo-siRNA function occurs in small, growing oocytes, and we utilized full-grown oocytes in our study, most likely the changes that we observe in the transcriptome are not only primary to disruption of siRNA function, but represent a complex array of downstream effects. Interrogating the transcriptome in growing oocytes should provide a better picture of the direct targets of endo-siRNAs.

**Fig 5:**
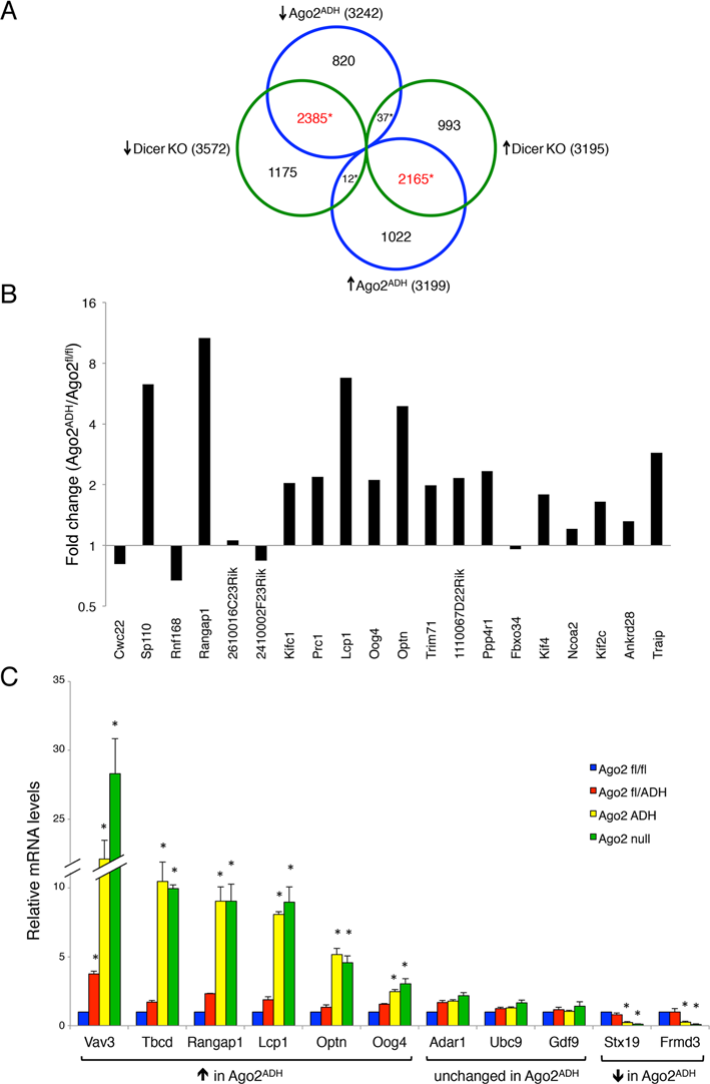
Extensive transcriptome changes, and high correlation with *Dicer* KO oocytes, in *Ago2* ^ADH^ oocytes. Oocytes from *Ago2* ^fl/fl^, *Ago2* ^ADH^, *Ago2* null, *Dicer* WT, and *Dicer* KO females were subjected to RNA-seq. A) Comparison of transcripts up-regulated (↑) or down-regulated (↓) in *Ago2* ^ADH^ vs. *Ago2* ^fl/fl^ oocytes (blue circles) with those up-regulated (↑) or down-regulated (↓) in *Dicer* KO vs. *Dicer* WT oocytes (green circles). Mis-regulated transcripts were identified using an FDR of 0.01. The overlapping transcripts are shown in red. *p< 2.2e-16, Chi-square test. B) The majority of genes that produce endo-siRNAs in oocytes are up-regulated in the absence of AGO2 catalytic activity. Transcript levels in our RNA-seq dataset were compared in *Ago2* ^ADH^ vs. *Ago2* ^fl/fl^ oocytes for the 20 genes that produce the largest number of endo-siRNAs [9] and fold-changes were calculated. C) Validation of RNA-seq data by qRT-PCR. The relative abundance of 11 selected transcripts [6 up-regulated (↑), 3 unchanged, and 2 down-regulated (↓)] in our RNA-seq dataset when comparing *Ago2* ^ADH^ vs. *Ago2* ^fl/fl^ oocytes) was determined in oocytes of the different *Ago2* genotypes by qRT-PCR. Transcript levels in *Ago2* ^fl/fl^ oocytes were set as 1. Data are expressed as the mean ± SEM of 3 experiments. *p< 0.05 vs. *Ago2* ^fl/fl^; two-way ANOVA, followed by Bonferroni post-test.

As expected, there was an excellent correlation between the *Ago2* ^ADH^ and *Dicer* datasets (Fig. 5A). Of the 3242 transcripts that were down-regulated in *Ago2* ^ADH^ vs. *Ago2* ^fl/fl^ oocytes, 2385 (74%) were also down-regulated in *Dicer* KO vs. WT oocytes. Similarly, of the 3199 transcripts that were up-regulated in *Ago2* ^ADH^ vs. *Ago2* ^fl/fl^ oocytes, 2165 (68%) were also up-regulated in *Dicer* KO vs. WT oocytes. Comparable numbers were obtained when *Ago2* null and *Dicer* datasets were compared (S4 Fig.). Also as expected, the transcriptome of *Ago2* ^ADH^ and *Ago2* null oocytes was very similar, with only 33 transcripts (24 genes) whose abundance differs between these two groups, one of them being *Ago2* itself (S5 Fig., S1 Table). Accordingly, the overlap between genes up-regulated compared to *Ago2* ^fl/fl^ oocytes in both groups or down-regulated in both groups is quite high (79–84%, S6 Fig.).

Although the overlap between genes mis-regulated in *Ago2* ^ADH^ and *Dicer* KO oocytes is quite high, there are many genes that are regulated differently in both groups. One possible explanation for these differences is that endo-siRNAs could have additional functions not mediated through AGO2-dependent endonucleolytic cleavage of target mRNAs. Also, AGO2 could cleave other, yet uncharacterized, DICER-independent small RNAs.

Given that a population of endo-siRNAs in oocytes derives from protein-coding genes, it was postulated that these small RNAs regulate expression of their precursor mRNAs [9,10]. To test this hypothesis, we analyzed our RNA-seq data for the transcript levels of the 20 genes that produce the largest number of siRNAs in oocytes [9]. The vast majority (15/20) are up-regulated in the absence of AGO2 catalytic activity (Fig. 5B), demonstrating a functional role for endo-siRNAs in the regulation of endogenous transcripts through endonucleolytic cleavage. The RNA-seq data were validated by performing qRT-PCR on several transcripts for which expression was either significantly increased, decreased, or unchanged in *Ago2* ^ADH^ oocytes, obtaining very similar results (Fig. 5C). We had previously demonstrated that the transcripts levels of genes that make siRNAs were increased in *Dicer* null oocytes [9], indicating a gene regulatory role for these small RNAs. Nevertheless, it was not clear if transcript regulation was due to endonucleolytic cleavage or if the mere production of siRNAs was diminishing the relative abundance of the transcript. Our results demonstrate that the action of siRNAs is through endonucleolytic cleavage of target mRNAs.

To gain insight into specific pathways that could be affected in *Ago2* ^*ADH*^ oocytes, gene ontology analysis of mis-regulated transcripts was performed using the database for annotation, visualization and integrated discovery (DAVID). For genes that are up-regulated in *Ago2* ^*ADH*^ oocytes, cell cycle, cell division, and regulation of translation, as well as microtubules and ribosomes were enriched (S7 Fig.); very similar categories were over-represented among genes up-regulated in *Dicer* KO oocytes (S8 Fig.). Many more categories were enriched among the genes that are down-regulated in *Ago2* ^*ADH*^ oocytes (S9 Fig.); these include RNA binding, nucleotide binding, cell cycle, chromosome, and transcription. And there was also an excellent correlation with those categories enriched for genes that are down-regulated in *Dicer* KO oocytes (S10 Fig.).

Although the miRNA pathway is dispensable in mouse oocytes, we were interested in determining if miRNA levels were normal in *Ago2* ^ADH^ oocytes, because *Ago2* null oocytes have reduced miRNA levels [15]. The concentration of 5 abundant miRNAs was assayed in oocytes of different *Ago2* genotypes. Mature miRNA levels were significantly decreased in both *Ago2* ^ADH^ and *Ago2* null oocytes (S11A Fig.). Consistent with this finding, the modest miRNA-mediated translational repression, as assayed using luciferase reporters, was also reduced (S11B Fig.). Although AGO proteins stabilize mature miRNAs (and hence AGO loss leads to miRNA turnover), the catalytic activity of AGO2 is not required for this effect [19–21]. There are at least two possible explanations for the discrepancy with our results. First, *Ago2* ^ADH^ oocytes contain only one allele of *Ago2* and hence the amount of protein is likely half the amount present in wild-type oocytes. Although *Ago3* is the most abundant *Ago* transcript in mouse oocytes (S12A Fig.), *Ago2* levels are substantial and a decrease in available AGO protein concentration may affect miRNA stability. Also, the levels of the other *Ago* transcripts are unchanged in both *Ago2* ^ADH^ and *Ago2* null oocytes (S12B Fig.; only *Ago2* and *Ago3* transcript levels are shown because *Ago1* and *Ago4* mRNA levels are extremely low, undetectable in many samples, but are not up-regulated in *Ago2* ^ADH^ oocytes). Second, the aforementioned studies were performed in somatic cells, which lack endo-siRNAs. Because the catalytic activity of AGO2 is required for passenger strand cleavage and siRNA unwinding [22–25], in *Ago2* ^ADH^ oocytes siRNA duplexes would remain associated with AGO2, preventing miRNA binding and thus leading to more rapid miRNA turnover. The *Zp3*-driven Cre recombinase utilized to delete the floxed allele of *Ago2* is active very early during oocyte growth [26], which takes ~ 3 weeks during which time transcription starts to decrease around mid-growth such that the full grown oocyte is transcriptionally inactive. Therefore, a small difference in miRNA stability can result over time in a highly significant decrease in miRNA levels. However, because mice whose oocytes are depleted of miRNAs show no discernable phenotype [12], the phenotype of *Ago2* ^ADH^ mice cannot be attributed to differences in oocyte miRNA levels.

In mammals, endo-siRNAs have only been described in mouse oocytes, ES cells, and male germ cells [9,10,27,28]. A physiological role for endo-siRNAs, however, has not been demonstrated in mammals. Mouse oocytes and ES cells lack the interferon response, an anti-viral defense mechanism against long dsRNA [29,30], and germ cells in the testis have also been suggested to be insensitive to interferon and hence tolerate dsRNA precursors that could generate endo-siRNAs [28]. In the mouse testis, ablation of *Dicer* or *Drosha* in germ cells leads to abnormal spermatogenesis, but male mice with a germ cell-specific ablation of *Ago2* show no phenotype [31,32], suggesting that miRNAs are essential for spermatogenesis, but endo-siRNAs are dispensable in the male germline. In contrast, we find that endo-siRNAs are essential in the female germline in mouse. The presence in oocytes of *DICER* ^*O*^ that efficiently generates siRNAs from long dsRNA precursors, coupled with the absence of an interferon response, makes the mouse oocyte a privileged environment for siRNA action and may explain why this highly specialized cell relies on the siRNA pathway to regulate gene expression and protect genomic integrity. Given that *DICER* ^*O*^ is only expressed in mouse and rat oocytes, but not other rodent or non-rodent species [13], this essential role of siRNAs in oocytes may be restricted to the *Muridae* family.

Because most animal miRNAs silence their targets by translational repression, often linked to mRNA decay, but not by endonucleolytic cleavage, it has been puzzling that one mammalian AGO protein (AGO2) has retained catalytic activity. The finding that the catalytic activity of AGO2 is required for biosynthesis of one miRNA, miR-451 [14], and that this small RNA is essential for erythropoiesis [33] provided an answer to this conundrum. Our findings of an essential role of siRNAs through endonucleolytic cleavage during female meiosis strengthen the idea of evolutionary pressure that at least one AGO retain catalytic activity.

## Materials and Methods

### Animals

*Ago2* ^fl/+^ animals [20] were crossed to *Ago2* ^ADH/+^ mice [14]. The resulting *Ago2* ^fl/ADH^ females were crossed to Zp3-Cre males (Jackson Laboratories) and their progeny were intercrossed to produce *Ago2* ^fl/ADH^; Cre/+ (*Ago2* ^ADH^) mice (S1 Fig.). These crosses also generated *Ago2* null mice. To determine fertility, two *Ago2* ^ADH^ and two *Ago2* ^fl/ADH^ female mice were bred with several males of proven fertility for a period of 6 months. Oocyte-specific *Dicer* null mice have been described [7]. All animal experiments were approved by the Institutional Animal Use and Care Committee of the University of Pennsylvania (protocol number 803551) and were consistent with National Institutes of Health guidelines.

### Oocyte collection, meiotic maturation, and culture

Four- to six-week-old female mice were primed by intraperitoneal injection of 5 IU of equine chorionic gonadotropin (eCG) 48 h before oocyte collection. Full-grown, germinal vesicle (GV)-intact cumulus-enclosed oocytes were collected as previously described [34]. The collection medium was bicarbonate-free minimal essential medium (Earle’s salt) supplemented with polyvinylpyrrolidone (3 mg/mL) and 25 mM HEPES, pH 7.3 (MEM/PVP). Germinal vesicle breakdown was inhibited by including 2.5 μM milrinone [35]. The oocytes were transferred to CZB medium [36] containing 2.5 μM milrinone and cultured in an atmosphere of 5% CO_2_ in air at 37°C until microinjection was performed. In experiments in which oocyte maturation was assessed, after collection the oocytes were transferred to milrinone-free CZB medium and cultured for 16h in an atmosphere of 5% CO_2_ in air at 37°C.

### Oocyte microinjection

GV oocytes were microinjected with approximately 5 pL of either siRNAs or cRNAs in MEM/PVP containing 2.5 μM milrinone as previously described [37]. *c-Mos* siRNA (CTGAACATTGCAAGACTAC; Dharmacon) was microinjected at 50 μM. For live imaging experiments, oocytes were microinjected with *Aurka-Gfp* cRNA (590 ng/μl) and *H2b-mCherry* cRNA (1035 ng/μL). miRNA reporters and firefly luciferase cRNAs were microinjected at 0.05 μg/μl.

### Immunohistochemistry, immunofluorescence and live cell imaging

For immunohistochemistry, whole ovaries were fixed for 16h in Bouin’s fixative, embedded in paraffin, sliced to 10-μm sections, and stained with hematoxylin and eosin.

Immunofluorescence was performed as previously described [38]. The meiotic spindle was stained with a mouse anti-(-tubulin monoclonal antibody conjugated to AlexaFluor 488 (1:100; Life Technologies), the cortical actin cap was visualized with Alexa Fluor 633-conjugated phalloidin (1:500; Life Technologies). DAPI (Sigma) and TO-PRO3 (Life Technologies), both at 1.5 μg/mL, were used to label DNA and were added to the mounting medium (Vectashield, Vector Laboratories).

cRNAs encoding AURKA-GFP and H2B-mCherry were synthesized as described [39]. Oocytes were microinjected with *Aurka-Gfp* and *H2b-mCherry* cRNAs, cultured for 5 h in CZB + milrinone, and then transferred to individual drops of milrinone-free CZB medium, where meiotic maturation was assessed through live imaging, as described [39]. Images of individual cells were acquired every 18 min during 16 h and processed using NIH ImageJ software.

### mRNA quantitative RT—PCR

Total RNA was extracted from 20 full-grown oocytes using Trizol (Life Technologies), according to the manufacturer’s protocol, except that 2 ng of *Egfp* RNA was added to the Trizol at the beginning of RNA isolation to serve as an exogenous normalization gene. cDNA was prepared by reverse transcription of total RNA with Superscript II and random hexamer primers. One oocyte equivalent of the resulting cDNA was amplified using TaqMan probes and the ABI Prism Sequence Detection System 7000 (Applied Biosystems). Two replicates were run for each real-time PCR reaction; a minus template served as control. Quantification was normalized to *Egfp* within the log-linear phase of the amplification curve obtained for each probe/primer using the comparative *C*_T_ method (ABI PRISM 7700 Sequence Detection System, User Bulletin 2, Applied Biosystems, 1997). The TaqMan gene expression assays used were: Mm00445082_m1 (*Vav3*), Mm00551650_m1 (*Tbcd*), Mm00441071_m1 (*Rangap1*), Mm00620601_m1 (*Oog4*), Mm00786153_s1 (*Lcp1*), Mm00725286_m1 (*Optn*), Mm00433565_m1 (*Gdf9*), Mm00508001_m1 (*Adar1*), Mm00459008_m1 (*Stx19*), Mm00556276_m1 (*Frmd3*), Mm00462977_m1 (*Ago1*), Mm03053414_g1 (*Ago2*), Mm01188534_m1 (*Ago3*), and Mm00462659_m1 (*Ago4*). For *Ubc9*, *Egfp*, *and c-Mos*, custom TaqMan Gene Expression Assays were used that had the following primers and probes: *Ubc9* forward primer 5′-CAGGTGAGAGCCAAGGACAAA-3′, *Ubc9* reverse primer 5′-GGCCCACTGTACAGCTAACA-3′, *Ubc9* probe 5′-CTGGCCTGCATTGATC-3′; *Egfp* forward primer: 5′-GCTACCCCGACCACATGAAG-3′, *Egfp* reverse primer: 5′-CGGGCATGGCGGACTT-3′, *Egfp* probe: 5′-CAGCACGACTTCTTC-3′; *c-Mos* forward primer: 5′-GGGAACAGGTATGTCTGATGCA-3′, *c-Mos* reverse primer: 5′-CACCGTGGTAAGTGGCTTTATACA-3′, *c-Mos* probe: 5′-CCGAGCCAAACCCTC-3′.

### Transposon quantitative RT—PCR

RNA isolation and reverse transcription were performed as above. Real-time PCR was done using one oocyte equivalent per reaction and SYBR Green master mix. β-actin served as an internal control for normalization. Primer sequences were: MT.fwd: 5’-TGTTAAGAGCTCTGTCGGATGTTG-3’; MT.rev: 5’-ACTGATTCTT CAGTCCCAGCTAAC-3’; SineB1.fwd: 5’-GTGGCGCACGCCTTTAATC-3’; SineB1.rev: 5’-GACAGGGTTTCTCTGTGTAG-3’; SineB2.fwd: 5’-GAGATGGCTCAGTGGTTAAG-3’; SineB2.rev: 5’-CTGTCTTCAGACACTCCAG-3’; Line L1 ORF2.fwd: 5’-TTTGGGACACAATGAAAGCA-3’; Line L1 ORF2.rev: 5’-CTGCCGTCTACTCCTCTTGG-3’; IAP LTR.fwd: 5’-TTGATAGTTGTGTTTTAAGTGGTAAATAAA-3’; IAP LTR.rev: 5’-AAAACACCACAAACCAAAATCTTCTAC-3’; actin.fwd: 5’- CGGTTCCGATGCCCTGAGGCTCTT-3’; actin.rev: 5’-CGTCACACTTCATGATGGAATTGA-3’.

### miRNA quantitative RT—PCR

miRNA levels were assayed using the TaqMan MicroRNA Cells-to-C_T_ kit (Life Technologies), following the manufacturers’ instructions, with slight modifications. Briefly, 9.1 μl of lysis solution was added to a tube containing 50 previously frozen full-grown oocytes. The samples were incubated for 8 min at room temperature and then 0.9 μl of stop solution was added, followed by a 2 min incubation at room temperature. Reverse transcription was performed using MultiScribe reverse transcriptase and following a multiplex protocol where the different miRNA-specific primers are mixed at a final concentration of 250 nM each. The resulting cDNA was diluted 10 times and real-time PCR was performed as described for mRNAs, using *snoRNA202* as normalizing control. The following small RNA TaqMan assays were used: 000391 (*mmu-miR-16–5p*), 000580 (*mmu-miR-20a-5p*), 000602 (*mmu-miR-30b-5p*), 002459 (*mmu-miR-106a-5p*), 002406 (*mmu-let-7e-5p*), and 001232 (*snoRNA202*).

### RNA sequencing

Twenty oocytes were lysed in 5 μL of NuGen lysis buffer. Each tube contained oocytes derived from 3 or 4 different animals of the same genotype and collection was performed three times to obtain 3 replicates per group. The groups were: *Ago2* ^fl/fl^, *Ago2* ^ADH^, *Ago2* null, *Dicer* WT and *Dicer* KO. The lysate was used for cDNA synthesis using the Ovation RNA-Seq System V2 (Nugen) according to the manufacturer’s protocol. The resulting cDNA was fragmented into 200bp using Covaris shearing, and the Ovation Ultralow DR Multiplex System (Nugen) was used for library construction. The size and concentration of the resulting libraries were checked on Bioanalyzer, quantified by qPCR and sequenced on Illumina HiSeq 2000 with PE50. Sequencing reads were mapped to the mm10 refGene transcriptome and genome using TopHat v2.0.3 [40] with options ‘--read-mismatches 1 --read-gap-length 1 --read-edit-dist 1 --max-multihits 100 --no-discordant --b2-very-sensitive --transcriptome-max-hits 100 --library-type fr-unstranded --no-coverage-search --no-novel-juncs’ for 36bp reads and ‘--read-mismatches 3 --read-edit-dist 3—max-multihits 100 --b2-very-sensitive --transcriptome-max-hits 100 --library-type fr-unstranded --no-coverage-search --no-novel-juncs’ for 50bp reads. Read counts were computed using htseq-count (http://dx.doi.org/10.1101/002824) with options ‘--stranded = no -mode = intersection-strict’. Differential expression analysis was performed using the DESeq R package (version 1.10.1) [41]. Gene ontology (GO) analysis was performed using the Database for Annotation, Visualization, and Integrated Discovery (DAVID) online resource [42,43] and using only the molecular function, cellular component, and biological process terms in the gene ontology database. The RNA-seq data have been deposited in NCBI’s Gene Expression Omnibus and are accessible through GEO Series accession number GSE57514 (http://www.ncbi.nlm.nih.gov/geo/query/acc.cgi?acc=GSE57514).

### Luciferase assays

Oocytes were microinjected with reporters that contain four bulged miR-30c sites (RL-4xB) downstream of the Renilla luciferase coding sequence. As a control, a reporter where the four miR-30c sites were mutated (RL-4xM) was used [11,44]. For normalization, firefly luciferase cRNA was coinjected with the Renilla luciferase reporters. The experiments were performed as previously described [11].

### Statistical analysis

All experiments were replicated at least three times, except for luciferase assays, which were performed twice. Data were analyzed by ANOVA, followed by Bonferroni post-test. RNA-seq data were analyzed using a Chi-square test. A p< 0.05 was considered statistically significant.

## Supporting Information

S1 Fig Breeding scheme to generate mice with catalytically inactive AGO2 in their oocytes. *Ago2* ^fl/+^ animals were mated with *Ago2* ^ADH/+^ mice. The resulting *Ago2* ^fl/ADH^ females (black circle) were mated with mice carrying Cre recombinase under the control of the oocyte-specific *Zp3* promoter to achieve deletion of the floxed allele exclusively in oocytes. *Ago2* ^fl/+^; Cre/+ animals derived from this cross (blue circle) were crossed to *Ago2* ^fl/ADH^ animals. This cross generated an F3 that contained all 4 genotypes utilized in this study: *Ago2* ^fl/fl^ (fl/fl), *Ago2* ^fl/ADH^ (fl/ADH), *Ago2* ^fl/ADH^; Cre/+ (ADH, red circle), and *Ago2* ^fl/fl^; Cre/+ (null) mice.
(TIF)

S2 Fig Increased abundance of mouse transcript (MT) retrotransposon in *Dicer* null oocytes. The levels of various transposons were determined by qRT-PCR in oocytes from *Dicer* WT or KO females, as described in Materials and Methods. Transposon levels in *Dicer* WT oocytes were set as 1. Data are expressed as the mean ± SEM of four experiments. *p< 0.001; two-way ANOVA, followed by Bonferroni post-test.
(TIF)

S3 Fig Comparison of magnitude of change in transcripts up-regulated vs. down-regulated between different RNAseq datasets. For each pair of samples, all transcripts that were differentially expressed at a 1% FDR were analyzed. The absolute values of fold changes (in logarithmic scale) were calculated. A) *Ago2* ^ADH^ vs. *Ago2* ^fl/fl^, B) *Ago2* null vs. *Ago2* ^fl/fl^, C) *Dicer* KO vs. WT. The differences between up-regulated and down-regulated transcripts for all three comparisons are significant (p< 2.2e-16 by a Wilcoxon rank-sum test).
(TIF)

S4 Fig Comparison of transcripts mis-regulated in *Ago2* null vs. *Dicer* KO oocytes. Comparison of transcripts up-regulated (↑) or down-regulated (↓) in *Ago2* null vs. *Ago2* ^fl/fl^ oocytes (blue circles) with those up-regulated (↑) or down-regulated (↓) in *Dicer* KO vs. *Dicer* WT oocytes (green circles). Mis-regulated transcripts were identified using an FDR of 1%. The overlapping transcripts are shown in red. *p< 2.2e-16, Chi-square test.
(TIF)

S5 Fig Analysis of differential expression of transcripts between *Ago2* ^ADH^ and *Ago2* null groups. The graph depicts the fold change (*Ago2* ^ADH^ vs. *Ago2* null) in a logarithmic scale versus expression levels. Each transcript is represented with a dot. Transcripts that are differentially expressed (FDR = 1%) are colored in red.
(TIF)

S6 Fig The transcriptomes of *Ago2* ^ADH^ and *Ago2* null oocytes are very similar. A) Overlap between transcripts up-regulated (↑) in *Ago2* ^ADH^ vs. *Ago2* ^fl/fl^ oocytes (blue circles) and those up-regulated (↑) in *Ago2* null vs. *Ago2* ^fl/fl^ oocytes (green circles). B) Overlap between transcripts down-regulated (↓) in *Ago2* ^ADH^ vs. *Ago2* ^fl/fl^ oocytes (blue circles) and those down-regulated (↓) in *Ago2* null vs. *Ago2* ^fl/fl^ oocytes (green circles). C) No overlap between transcripts up-regulated (↑) in *Ago2* ^ADH^ vs. *Ago2* ^fl/fl^ oocytes (blue circles) and those down-regulated (↓) in *Ago2* null vs. *Ago2* ^fl/fl^ oocytes (green circles). D) No overlap between transcripts down-regulated (↓) in *Ago2* ^ADH^ vs. *Ago2* ^fl/fl^ oocytes (blue circles) and those up-regulated (↑) in *Ago2* null vs. *Ago2* ^fl/fl^ oocytes (green circles). In all cases, mis-regulated transcripts were identified using an FDR of 1%. The overlapping transcripts are shown in red. *p< 2.2e-16, Chi-square test.
(TIF)

S7 Fig Gene ontology (GO) analysis of transcripts up-regulated in *Ago2* ^ADH^ vs. *Ago2* ^fl/fl^ oocytes. Up-regulated transcripts were identified using an FDR of 1% and analyzed using the functional annotation tool in DAVID. Only significant and non-redundant categories are shown (Benjamini p value< 0.05).
(TIF)

S8 Fig Gene ontology (GO) analysis of transcripts up-regulated in *Dicer* KO vs. *Dicer* WT oocytes. Up-regulated transcripts were identified using an FDR of 1% and analyzed using the functional annotation tool in DAVID. Only significant and non-redundant categories are shown (Benjamini p value< 0.05).
(TIF)

S9 Fig Gene ontology (GO) analysis of transcripts down-regulated in *Ago2* ^ADH^ vs. *Ago2* ^fl/fl^ oocytes. Down-regulated transcripts were identified using an FDR of 1% and analyzed using the functional annotation tool in DAVID. Only significant and non-redundant categories are shown (Benjamini p value< 0.05).
(TIF)

S10 Fig Gene ontology (GO) analysis of transcripts down-regulated in *Dicer* KO vs. *Dicer* WT oocytes. Down-regulated transcripts were identified using an FDR of 1% and analyzed using the functional annotation tool in DAVID. Only significant and non-redundant categories are shown (Benjamini p value< 0.05).
(TIF)

S11 Fig Decreased miRNA levels and miRNA activity in *Ago2* ^ADH^ oocytes. A) The levels of various abundant miRNAs in mouse oocytes were determined by qRT-PCR in oocytes from different *Ago2* genotypes, as described in Materials and Methods. miRNA levels in *Ago2* ^fl/fl^ oocytes were set as 1. Data are expressed as the mean ± SEM of four experiments. *p < 0.05 vs. *Ago2* ^fl/fl^; two-way ANOVA, followed by Bonferroni post-test. B) Relative Renilla luciferase activity in oocytes from *Ago2* ^*fl/*ADH^ and *Ago2* ^ADH^ mice. In vitro-transcribed reporter mRNAs containing four binding sites for miR-30c (RL-4xB) or a control reporter in which the miR-30c binding sites were mutated (RL-4xM) [11] were microinjected as described in Materials & Methods. Renilla luciferase reporter activities were normalized to the coinjected firefly luciferase control and are shown relative to the RL-4xM group, which was set to one. The experiment was performed twice, and similar results were obtained in each case. Shown are data (mean ± SEM) from one experiment. *p < 0.05 compared to control by one-way ANOVA, followed by Bonferroni post-test.
(TIF)

S12 Fig Relative abundances of Argonaute transcripts in mouse oocytes. A) Real-time RT-PCR of *Ago1*, *Ago2*, *Ago3*, and *Ago4* transcripts was performed in oocytes from *Ago2* ^*fl/fl*^ mice. Delta Rn is the magnitude of the fluorescence signal generated during PCR at each time point. The experiment was performed three times and a representative example is shown. B) Real-time RT-PCR of *Ago1*, *Ago2*, *Ago3*, and *Ago4* transcripts was performed in oocytes from different *Ago2* genotypes. *Ago1* and *Ago4* levels were either extremely low or undetectable; therefore, only *Ago2* and *Ago3* transcript levels are shown. Transcript levels in *Ago2* ^fl/fl^ oocytes were set as 1. Data are expressed as the mean ± SEM of three experiments. *p < 0.05 vs. *Ago2* ^fl/fl^; two-way ANOVA, followed by Bonferroni post-test.
(TIF)

S1 Table Transcripts differentially expressed between *Ago2* ^ADH^ and *Ago2* null oocytes. List of transcripts that were differentially expressed between *Ago2* ^ADH^ and *Ago2* null oocytes, using an FDR of 1%. A: *Ago2* null group, B: *Ago*2^ADH^ group. The fold change is calculated as base mean B/base mean A. Highlighted is the *Ago2* transcript.
(XLSX)

S1 Movie Live imaging of meiotic maturation in *Ago2* ^fl/ADH^ oocytes. The experiment was performed as described in Materials and Methods. AURKA-EGFP (green) labels the spindle poles and H2B-mCherry (red) labels chromosomes.
(AVI)

S2 Movie Live imaging of meiotic maturation in *Ago2* ^ADH^ oocytes. The experiment was performed as described in Materials and Methods. AURKA-EGFP (green) labels the spindle poles and H2B-mCherry (red) labels chromosomes.
(AVI)

S3 Movie Some *Ago2* ^ADH^ oocytes showed better chromosome alignment, but there were nonetheless some unaligned chromosomes and entry into anaphase failed. The experiment was performed as described in Materials and Methods. AURKA-EGFP (green) labels the spindle poles and H2B-mCherry (red) labels chromosomes.
(AVI)
