# Correction: Essential Role for Endogenous siRNAs during Meiosis in Mouse Oocytes

**DOI:** 10.1371/journal.pgen.1005129

**Published:** 2015-04-10

**Authors:** The PLOS Genetics Staff

The fifth author's name is spelled incorrectly. The correct name is: Olga Davydenko. The correct citation is: Stein P, Rozhkov NV, Li F, Cárdenas FL, Davydenko O, et al. (2015) Essential Role for Endogenous siRNAs during Meiosis in Mouse Oocytes. PLoS Genet 11(2): e1005013. doi:10.1371/journal.pgen.1005013
