# Supplementary figures and images for: Essential Role for Endogenous siRNAs during Meiosis in Mouse Oocytes
Source: PLoS Genet. 2015 Feb 19;11(2):e1005013. doi: 10.1371/journal.pgen.1005013 (PMC4335007; doi:10.1371/journal.pgen.1005013)

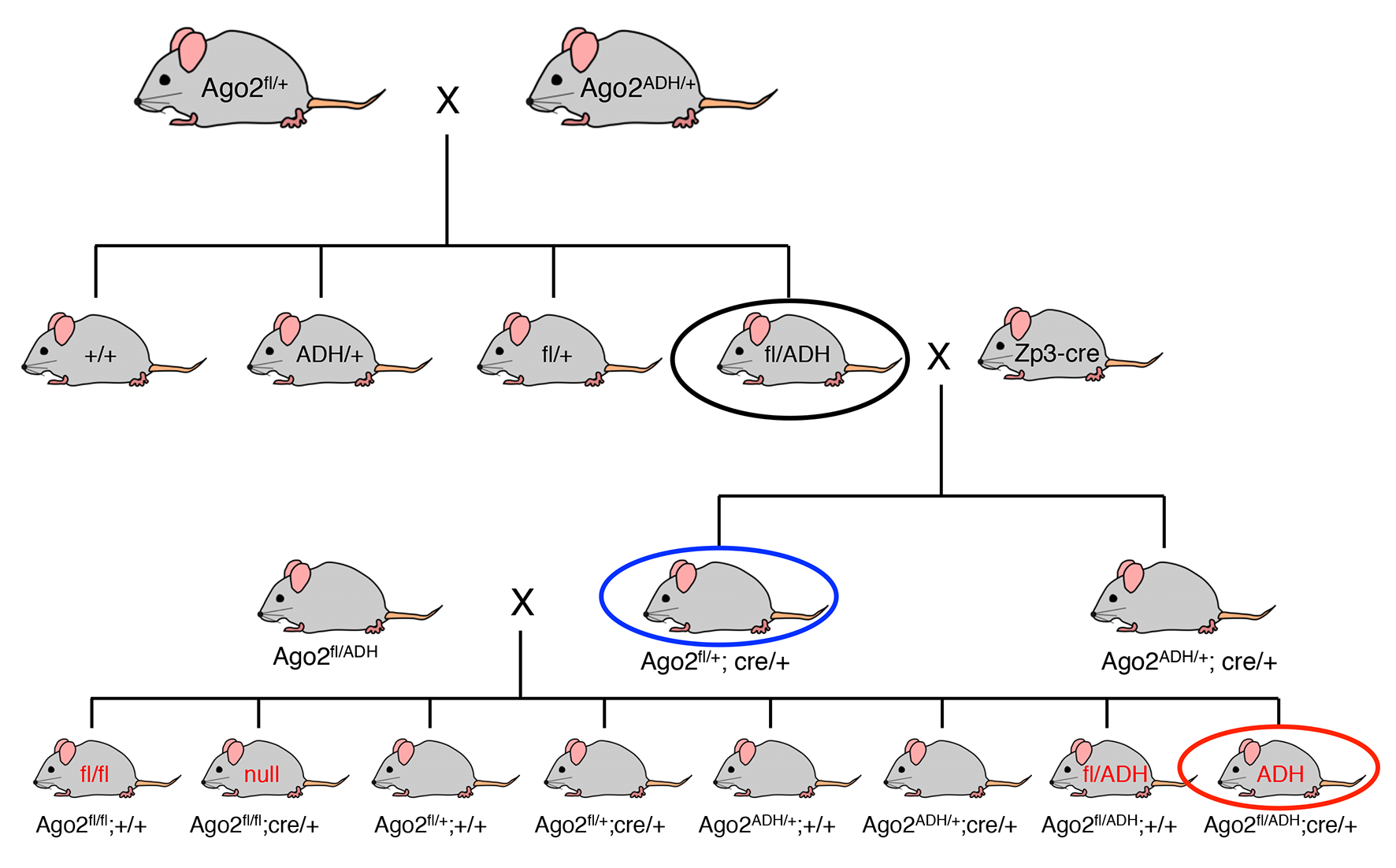

Supplement: S1 Fig — Ago2 fl/+ animals were mated with Ago2 ADH/+ mice. The resulting Ago2 fl/ADH females (black circle) were mated with mice carrying Cre recombinase under the control of the oocyte-specific Zp3 promoter to achieve deletion of the floxed allele exclusively in oocytes. Ago2 fl/+; Cre/+ animals derived from this cross (blue circle) were crossed to Ago2 fl/ADH animals. This cross generated an F3 that contained all 4 genotypes utilized in this study: Ago2 fl/fl (fl/fl), Ago2 fl/ADH (fl/ADH), Ago2 fl/ADH; Cre/+ (ADH, red circle), and Ago2 fl/fl; Cre/+ (null) mice. (TIF) [file pgen.1005013.s001.tif]

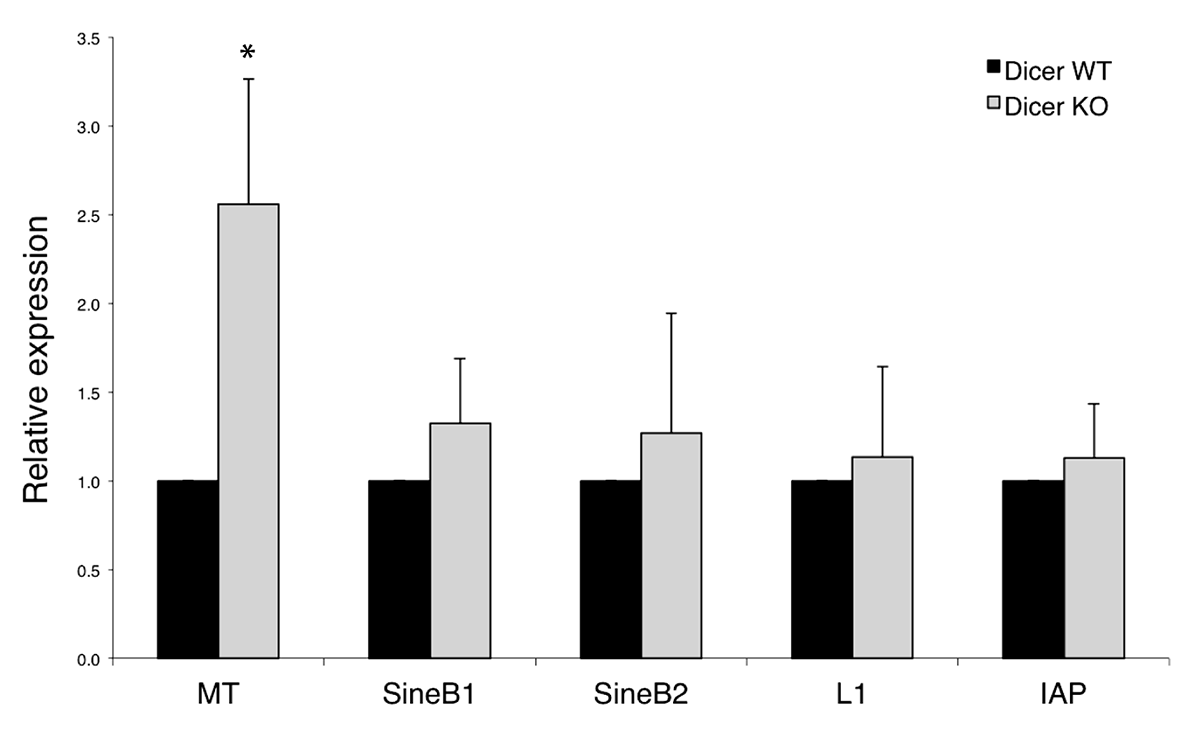

Supplement: S2 Fig — The levels of various transposons were determined by qRT-PCR in oocytes from Dicer WT or KO females, as described in Materials and Methods. Transposon levels in Dicer WT oocytes were set as 1. Data are expressed as the mean ± SEM of four experiments. *p< 0.001; two-way ANOVA, followed by Bonferroni post-test. (TIF) [file pgen.1005013.s002.tif]

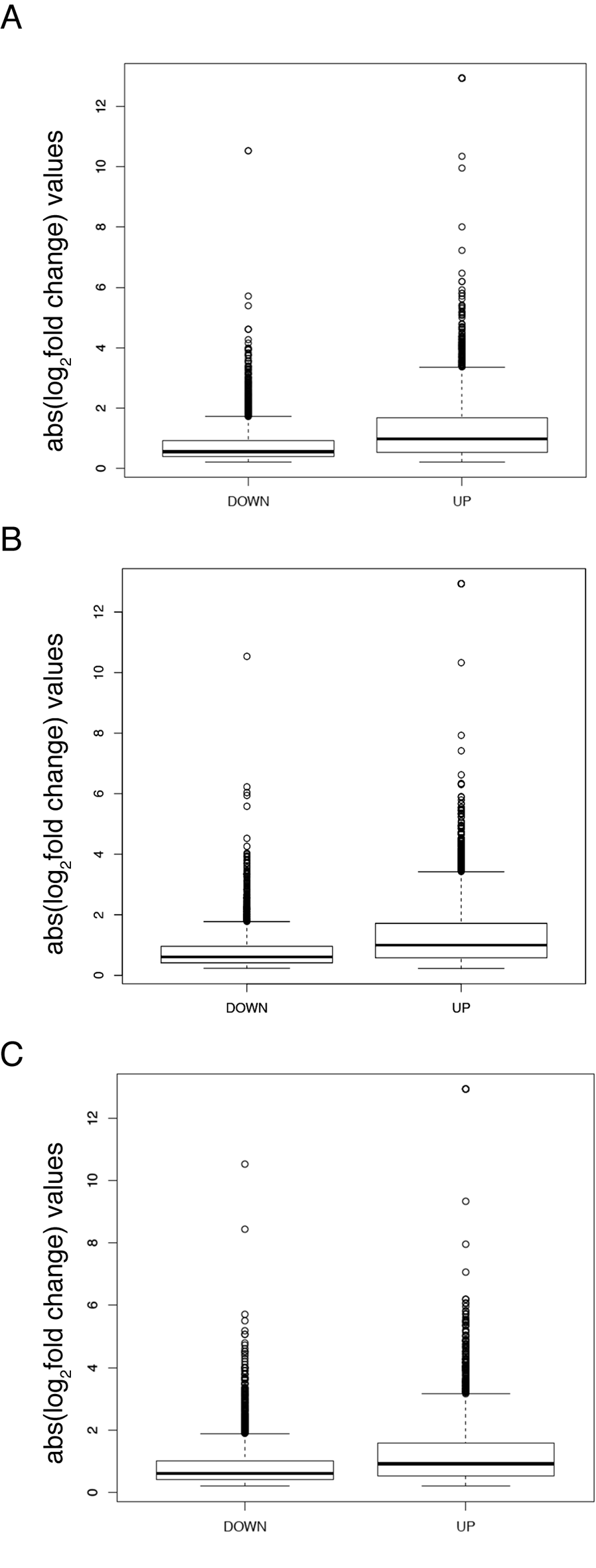

Supplement: S3 Fig — For each pair of samples, all transcripts that were differentially expressed at a 1% FDR were analyzed. The absolute values of fold changes (in logarithmic scale) were calculated. A) Ago2 ADH vs. Ago2 fl/fl, B) Ago2 null vs. Ago2 fl/fl, C) Dicer KO vs. WT. The differences between up-regulated and down-regulated transcripts for all three comparisons are significant (p< 2.2e-16 by a Wilcoxon rank-sum test). (TIF) [file pgen.1005013.s003.tif]

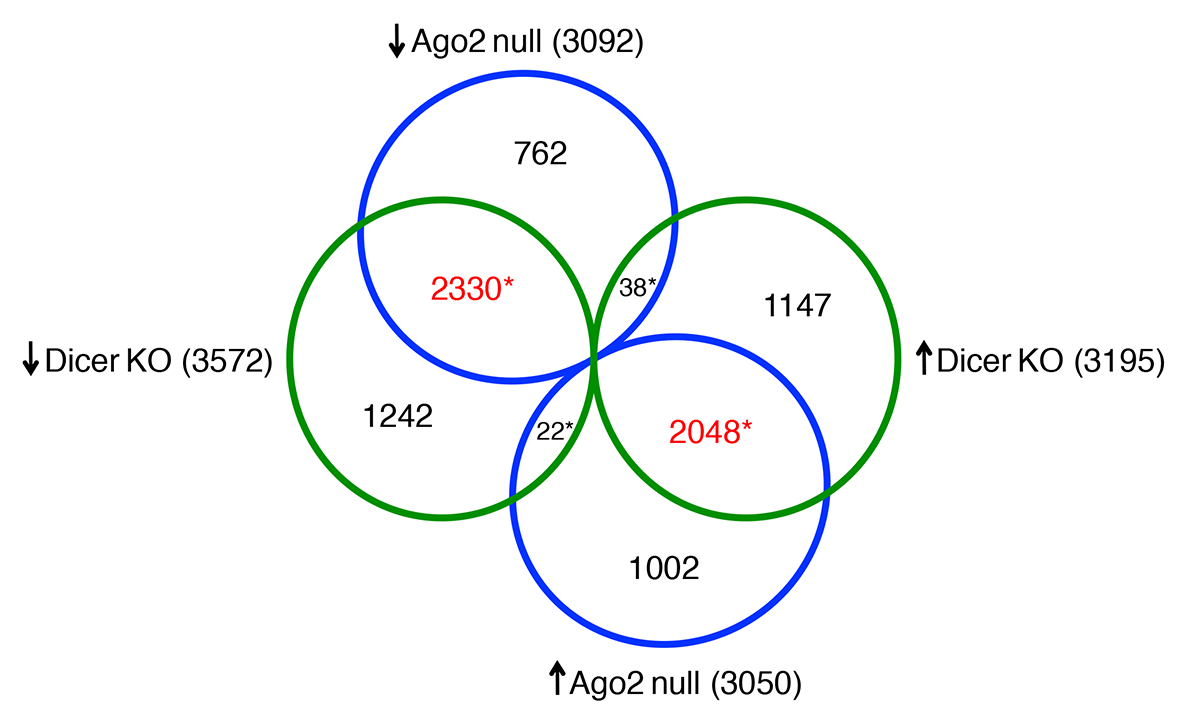

Supplement: S4 Fig — Comparison of transcripts up-regulated (↑) or down-regulated (↓) in Ago2 null vs. Ago2 fl/fl oocytes (blue circles) with those up-regulated (↑) or down-regulated (↓) in Dicer KO vs. Dicer WT oocytes (green circles). Mis-regulated transcripts were identified using an FDR of 1%. The overlapping transcripts are shown in red. *p< 2.2e-16, Chi-square test. (TIF) [file pgen.1005013.s004.tif]

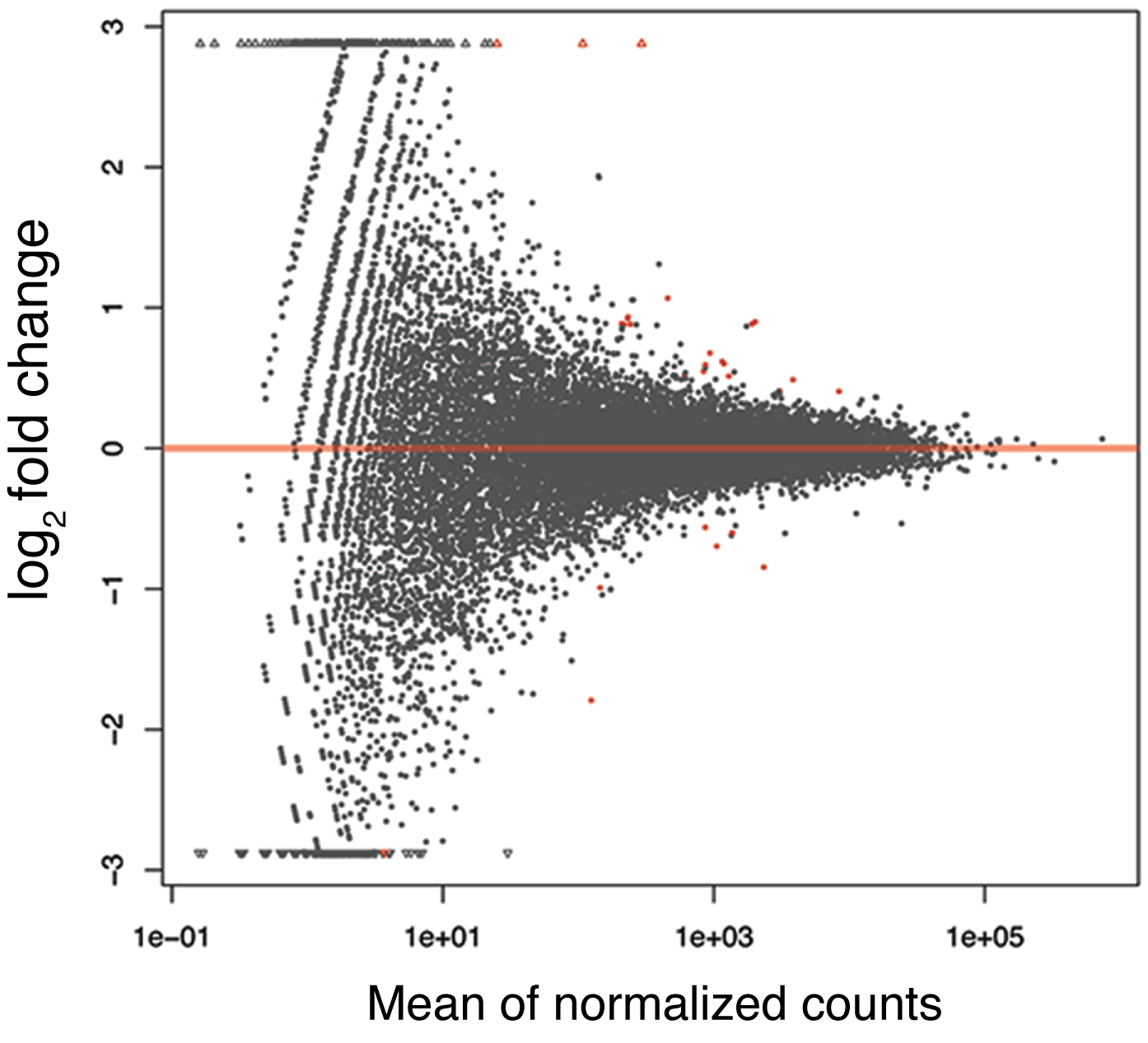

Supplement: S5 Fig — The graph depicts the fold change (Ago2 ADH vs. Ago2 null) in a logarithmic scale versus expression levels. Each transcript is represented with a dot. Transcripts that are differentially expressed (FDR = 1%) are colored in red. (TIF) [file pgen.1005013.s005.tif]

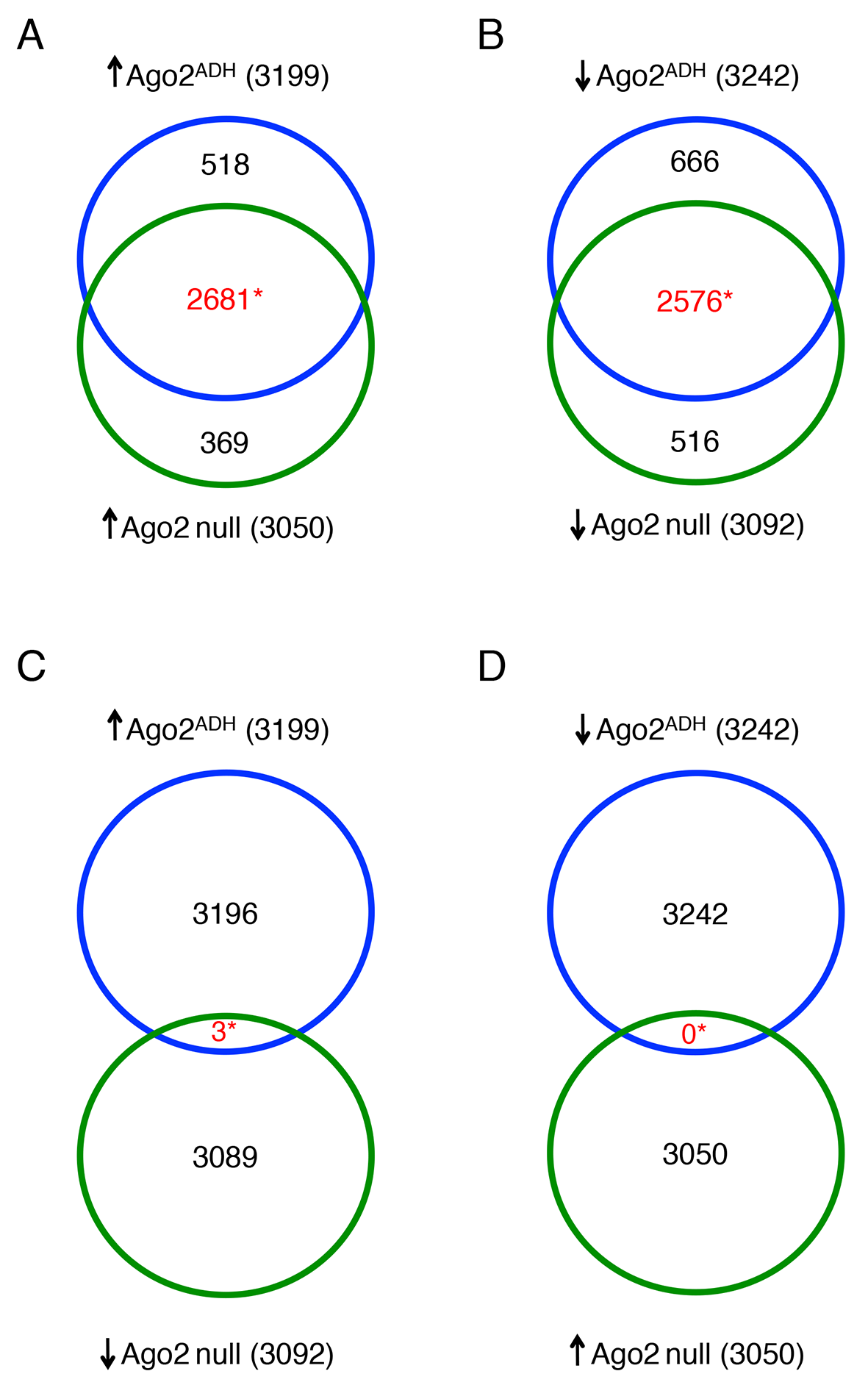

Supplement: S6 Fig — A) Overlap between transcripts up-regulated (↑) in Ago2 ADH vs. Ago2 fl/fl oocytes (blue circles) and those up-regulated (↑) in Ago2 null vs. Ago2 fl/fl oocytes (green circles). B) Overlap between transcripts down-regulated (↓) in Ago2 ADH vs. Ago2 fl/fl oocytes (blue circles) and those down-regulated (↓) in Ago2 null vs. Ago2 fl/fl oocytes (green circles). C) No overlap between transcripts up-regulated (↑) in Ago2 ADH vs. Ago2 fl/fl oocytes (blue circles) and those down-regulated (↓) in Ago2 null vs. Ago2 fl/fl oocytes (green circles). D) No overlap between transcripts down-regulated (↓) in Ago2 ADH vs. Ago2 fl/fl oocytes (blue circles) and those up-regulated (↑) in Ago2 null vs. Ago2 fl/fl oocytes (green circles). In all cases, mis-regulated transcripts were identified using an FDR of 1%. The overlapping transcripts are shown in red. *p< 2.2e-16, Chi-square test. (TIF) [file pgen.1005013.s006.tif]

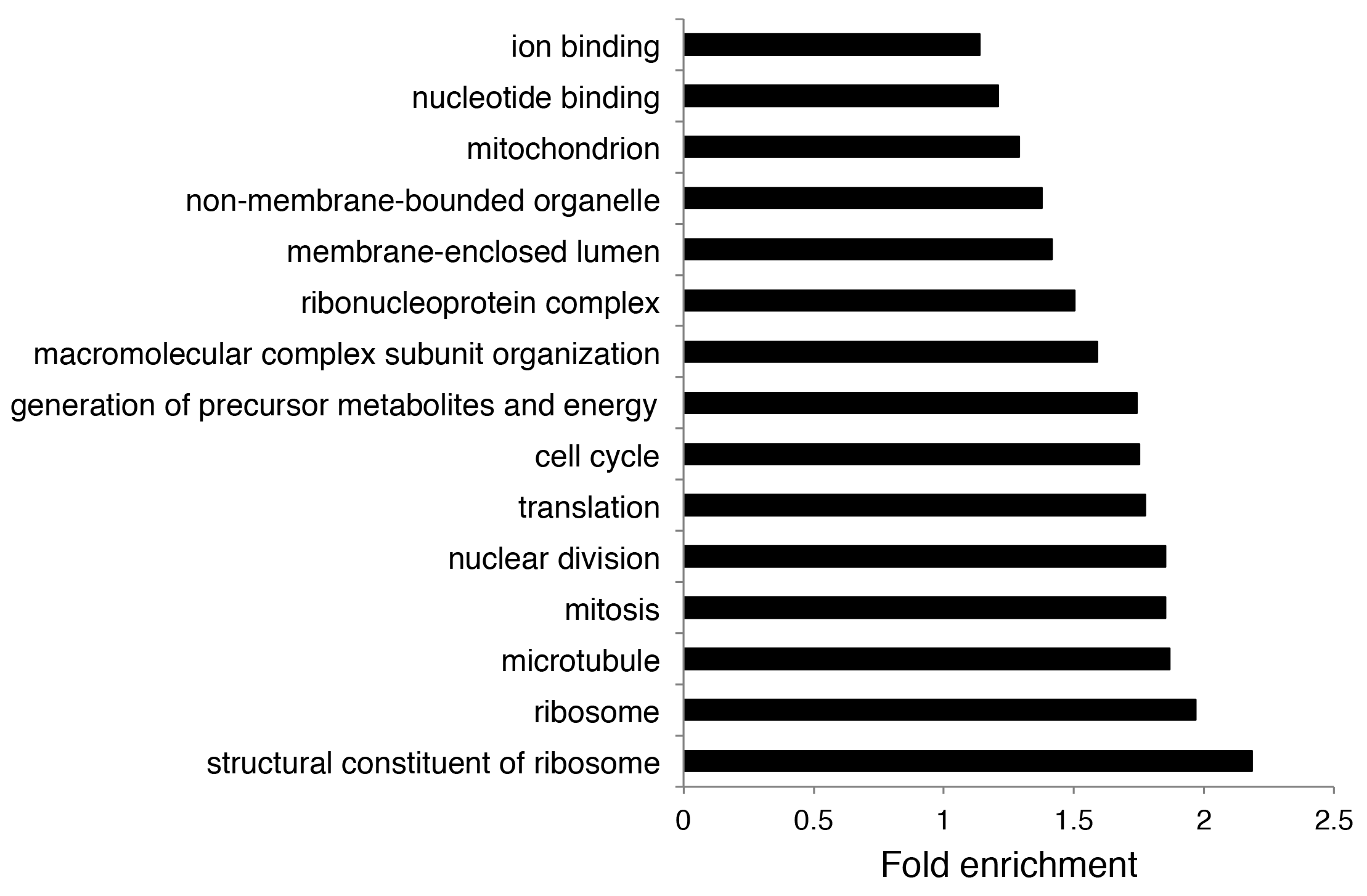

Supplement: S7 Fig — Up-regulated transcripts were identified using an FDR of 1% and analyzed using the functional annotation tool in DAVID. Only significant and non-redundant categories are shown (Benjamini p value< 0.05). (TIF) [file pgen.1005013.s007.tif]

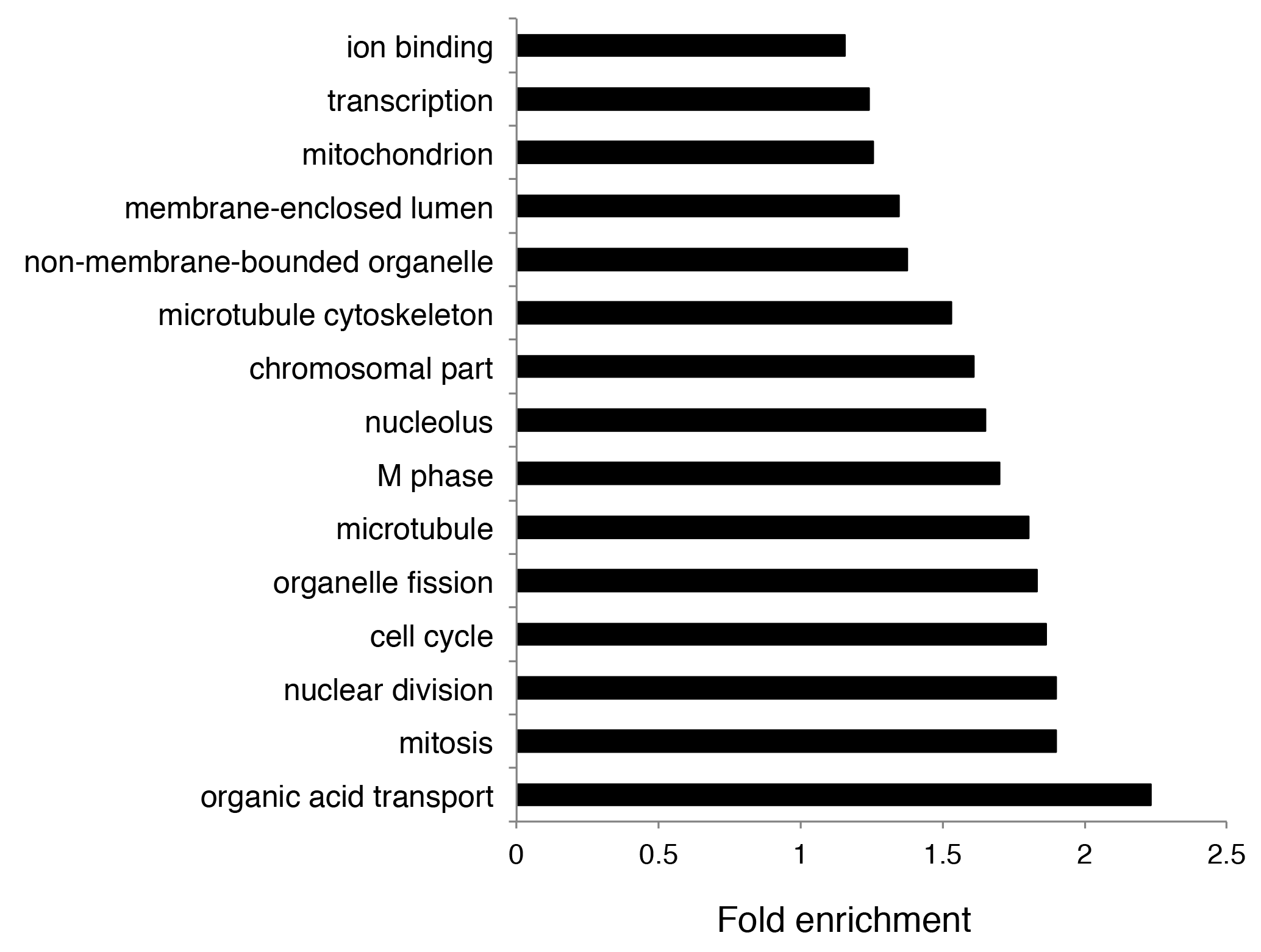

Supplement: S8 Fig — Up-regulated transcripts were identified using an FDR of 1% and analyzed using the functional annotation tool in DAVID. Only significant and non-redundant categories are shown (Benjamini p value< 0.05). (TIF) [file pgen.1005013.s008.tif]

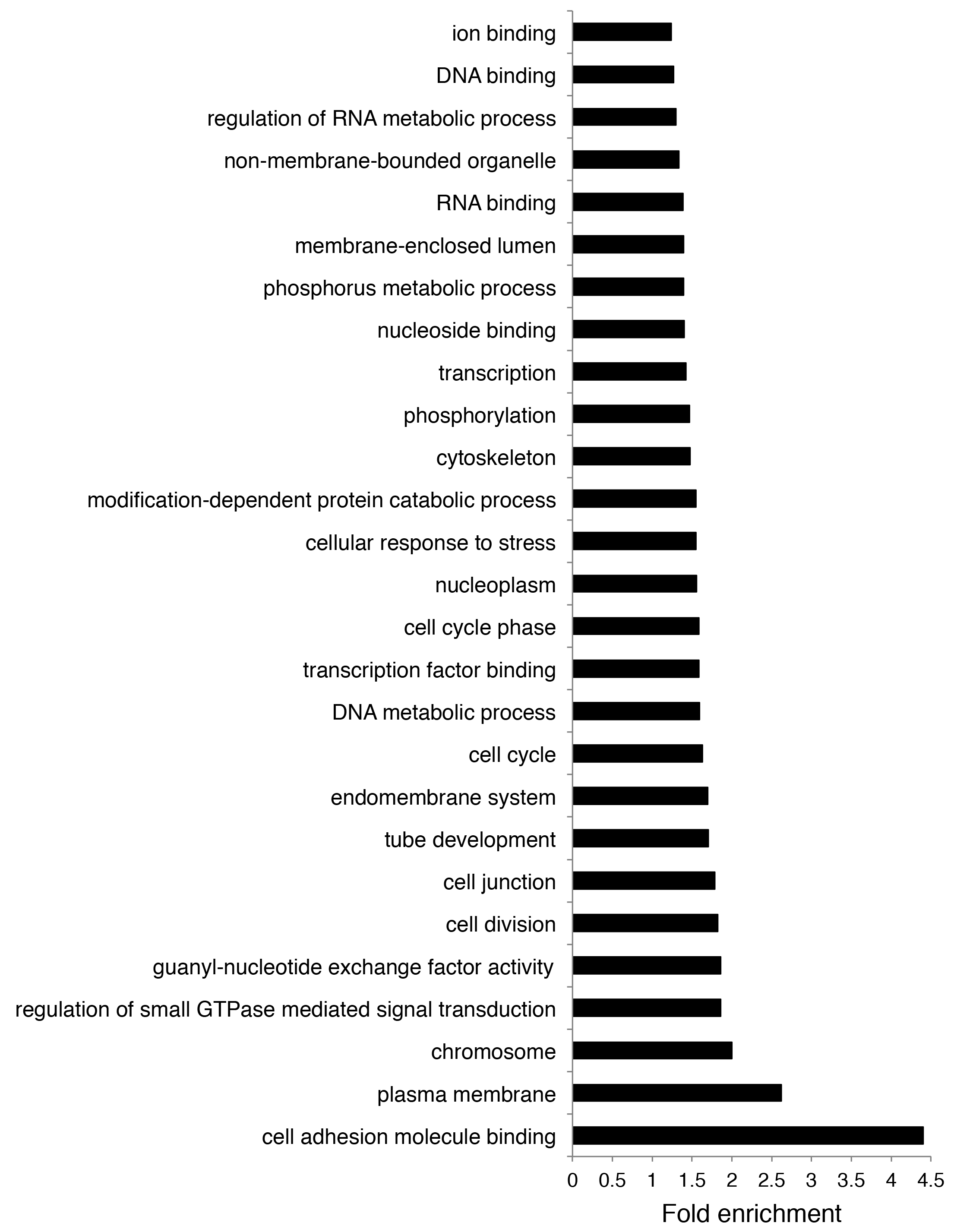

Supplement: S9 Fig — Down-regulated transcripts were identified using an FDR of 1% and analyzed using the functional annotation tool in DAVID. Only significant and non-redundant categories are shown (Benjamini p value< 0.05). (TIF) [file pgen.1005013.s009.tif]

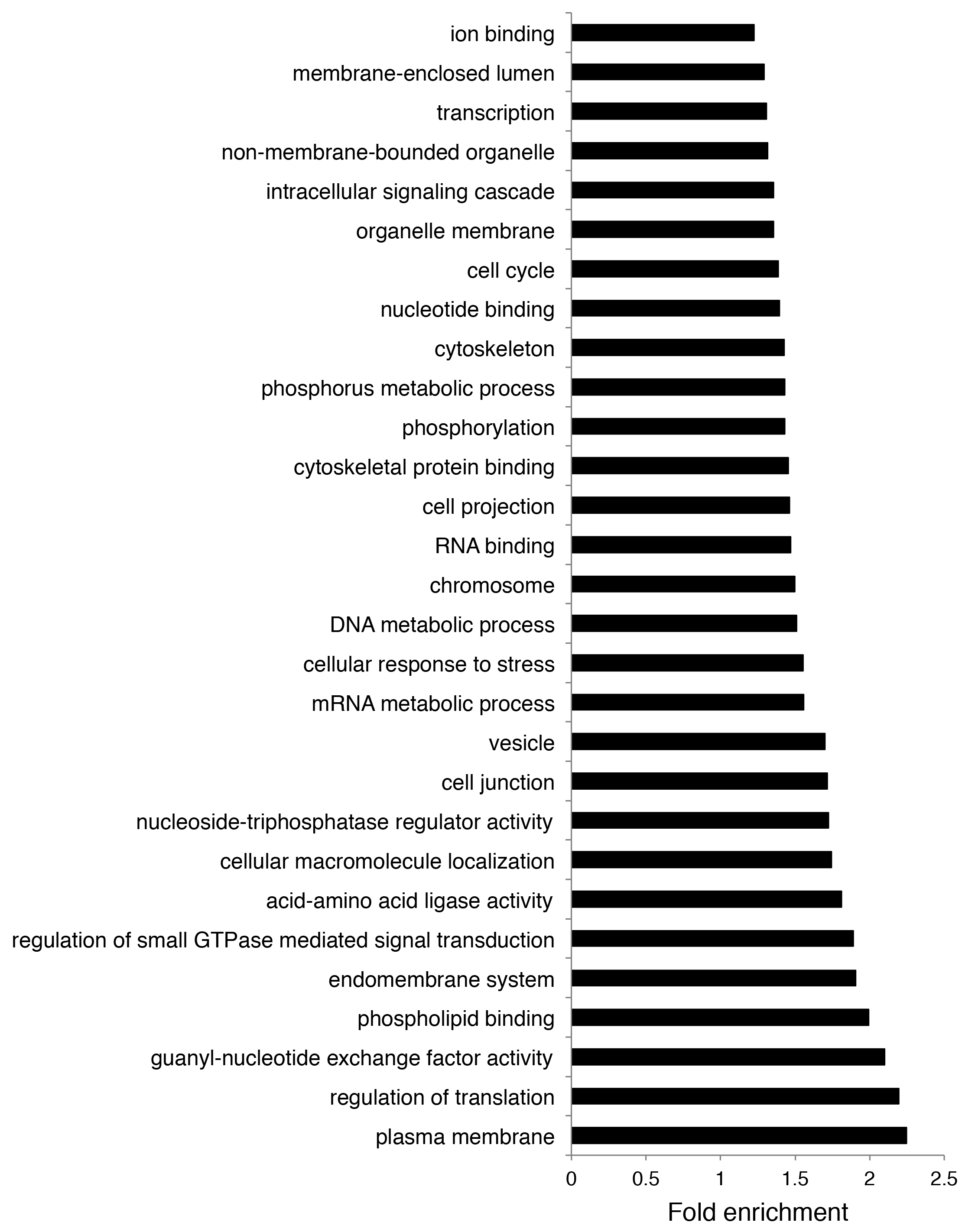

Supplement: S10 Fig — Down-regulated transcripts were identified using an FDR of 1% and analyzed using the functional annotation tool in DAVID. Only significant and non-redundant categories are shown (Benjamini p value< 0.05). (TIF) [file pgen.1005013.s010.tif]

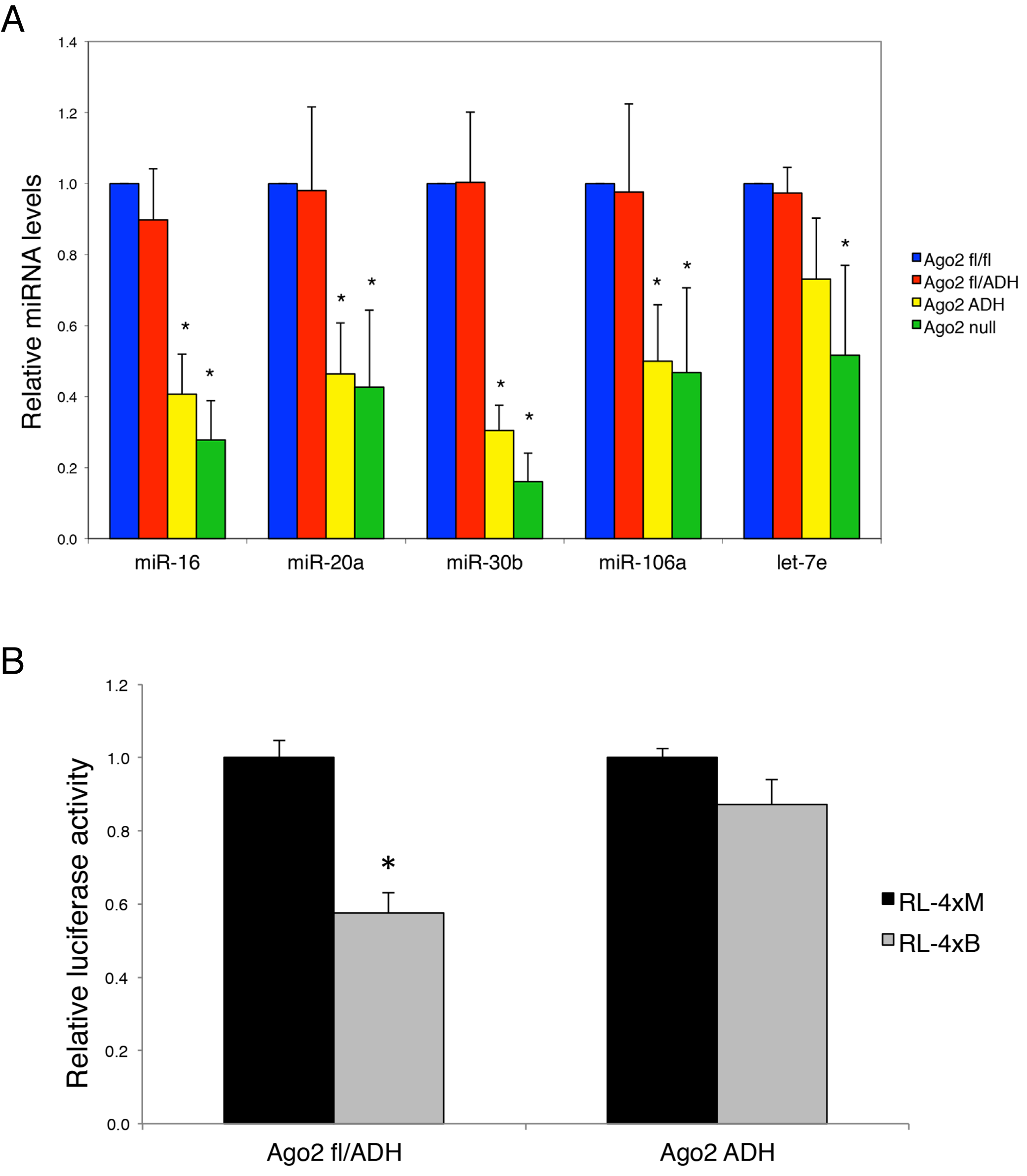

Supplement: S11 Fig — A) The levels of various abundant miRNAs in mouse oocytes were determined by qRT-PCR in oocytes from different Ago2 genotypes, as described in Materials and Methods. miRNA levels in Ago2 fl/fl oocytes were set as 1. Data are expressed as the mean ± SEM of four experiments. *p < 0.05 vs. Ago2 fl/fl; two-way ANOVA, followed by Bonferroni post-test. B) Relative Renilla luciferase activity in oocytes from Ago2 fl/ADH and Ago2 ADH mice. In vitro-transcribed reporter mRNAs containing four binding sites for miR-30c (RL-4xB) or a control reporter in which the miR-30c binding sites were mutated (RL-4xM) [11] were microinjected as described in Materials & Methods. Renilla luciferase reporter activities were normalized to the coinjected firefly luciferase control and are shown relative to the RL-4xM group, which was set to one. The experiment was performed twice, and similar results were obtained in each case. Shown are data (mean ± SEM) from one experiment. *p < 0.05 compared to control by one-way ANOVA, followed by Bonferroni post-test. (TIF) [file pgen.1005013.s011.tif]

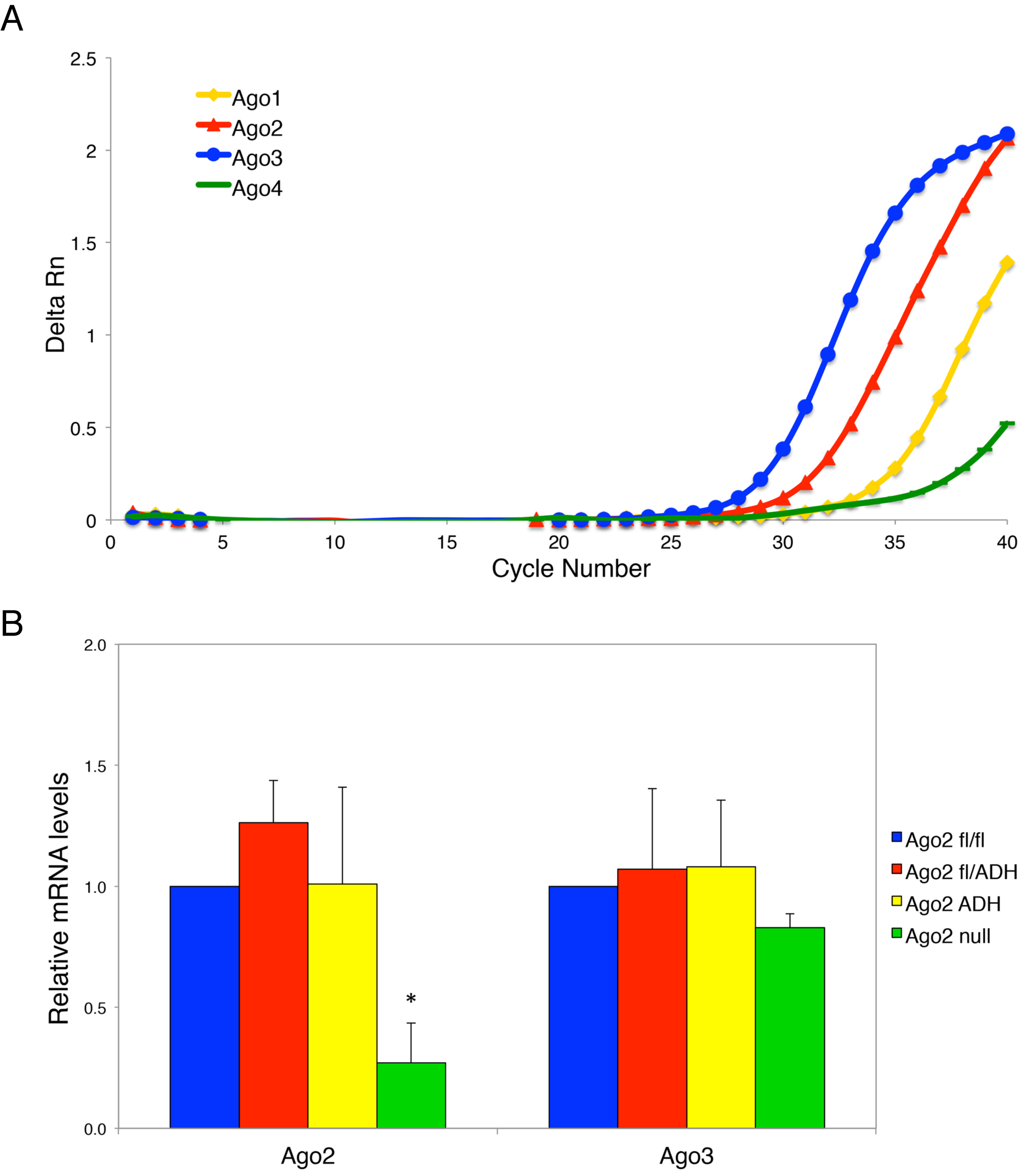

Supplement: S12 Fig — A) Real-time RT-PCR of Ago1, Ago2, Ago3, and Ago4 transcripts was performed in oocytes from Ago2 fl/fl mice. Delta Rn is the magnitude of the fluorescence signal generated during PCR at each time point. The experiment was performed three times and a representative example is shown. B) Real-time RT-PCR of Ago1, Ago2, Ago3, and Ago4 transcripts was performed in oocytes from different Ago2 genotypes. Ago1 and Ago4 levels were either extremely low or undetectable; therefore, only Ago2 and Ago3 transcript levels are shown. Transcript levels in Ago2 fl/fl oocytes were set as 1. Data are expressed as the mean ± SEM of three experiments. *p < 0.05 vs. Ago2 fl/fl; two-way ANOVA, followed by Bonferroni post-test. (TIF) [file pgen.1005013.s012.tif]
